# Enhancing the performance of solid-state supercapacitors using Li^+^-garnet polymer hybrids as electrolyte

**DOI:** 10.1039/d6ra05078f

**Published:** 2026-08-10

**Authors:** Kamaldeep Bisht, Bhargab Sharma, Hardeep, Arpita Mishra, Anshuman Dalvi

**Affiliations:** a Department of Physics, Birla Institute of Technology and Science Pilani Campus, Vidya Vihar Pilani Rajasthan 333031 India adalvi@pilani.bits-pilani.ac.in

## Abstract

The present work delves into the synthesis and characterization of Li^+^-ion garnet polymer composites and their novel electrolytic application in solid-state supercapacitors (SSCs). For this, Niobium (Nb) substituted cubic garnet LLZNO (Li_6.75_La_3_Zr_1.75_Nb_0.25_O_12_) was synthesized and reinforced into a PEO matrix, leading to an optimized composition of 12LiClO_4_ + 88{(PEO)_0.55_ (LLZNO)_0.45_}, with high ionic conductivity ∼2 × 10^−4^ Ω^−1^ cm^−1^ at 30 °C. Supercapacitors were then assembled using these electrolyte membranes with high surface-area (∼1800 m^2^ g^−1^) activated carbon electrodes. The SSC interface was subsequently tuned by forming a micro-gel layer at the electrode–electrolyte interface to achieve high performance parameters. For this, dimethylformamide (DMF) was incorporated in a minimal amount of ∼5 µL cm^−2^ at the interface. The SSCs with ∼3 V stability window exhibit high performance parameters, *viz.* specific capacitance ∼143 F g^−1^ at 2 V/1 mA, specific power ∼1303 W kg^−1^, and specific energy ∼24 Wh kg^−1^. The SSCs exhibit ∼60% and ∼53% capacitance retention after 15 000 and 100 000 galvanostatic charge–discharge cycles, respectively. The temperature tolerance of the SSCs was tested over a wide temperature range (−10 °C to 50 °C), and the performance parameters exhibit excellent reproducibility. Investigation reveals that these SSCs are predominantly of the electric-double-layer type. Finally, the practical use of fabricated SSCs is demonstrated by glowing two parallel-connected 3 V LEDs for ∼30 minutes.

## Abstract 2

The present work delves into the synthesis and characterization of Li^+^-ion garnet polymer composites and their novel electrolytic application in solid-state supercapacitors (SSCs).

## Graphical abstract

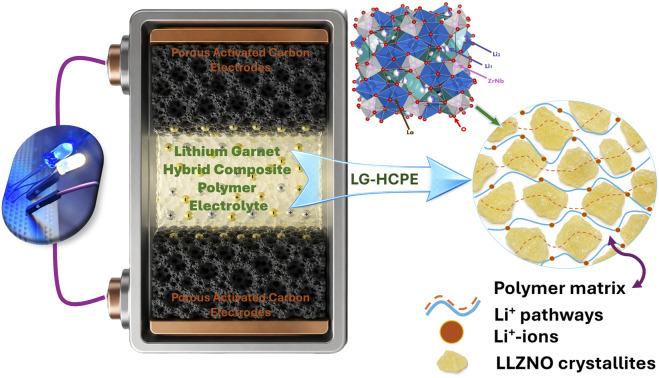

## 1 Introduction

A supercapacitor exhibits characteristics common to batteries and traditional capacitors.^1^ Its charge storage primarily occurs at the surface, enabling instantaneous charge transfer at the electrode–electrolyte interface, resulting in high power output. Depending on the electrolyte type, these are classified into liquid, gel, or solid-state supercapacitors.^2–4^ For a long time, liquid electrolytes have been used in traditional energy storage systems, but they pose safety risks due to thermal runaway and leakage. Various gel polymers have also been investigated for electrolytic applications in supercapacitors. Gel polymer electrolytes (GPEs) can overcome the limitations of liquid electrolytes by encapsulating them. The polymer matrix (PVA, PEO, PMMA, PVDF) traps the liquid content, offering a wider potential window, better safety, and greater flexibility.^5^ However, their thermal stability and mechanical strength are inadequate for commercial device applications. Different configurations of these gel-electrolytes, *i.e.* PVDF-HFP/IL/Li,^6^ PVA-H_2_SO_4_,^7^ PMMA-PC-LiClO_4_,^8^ show remarkable results. The reported performance for many gel-based devices lies between those of liquid and solid electrolytes, *i.e.* energy density (*E*) ∼10–50 Wh kg^−1^, power density (*P*) ∼500–10 kW kg^−1^, specific capacitance (*C*_S_) ∼100–300 F g^−1^, with cycle stability >20 000. Although liquid- and gel-based supercapacitors offer high performance, they still pose safety, mechanical stability and scalability challenges. Solid-state supercapacitors (SSCs) address these challenges and can also demonstrate superior performance when their interfaces are properly engineered.^9^ Moreover, unlike devices that use liquid electrolytes, SSCs can be scaled down to much lower dimensions.^10^

In principle, SSCs can also utilize fast-ionic conductors (FICs)^11^ with a conductivity ≥10^−4^ Ω^−1^ cm^−1^ near room temperature (RT). Several attempts have been made in recent years to develop ceramic-based SSCs,^12,13^ although their use in batteries has been well investigated for several decades. FICs, or ion-conducting ceramics such as NASICON,^14^ Perovskite,^15^ and Garnet^16^ are well known for their high ionic conductivity and wide electrochemical stability window. These properties make them advantageous as electrolytes, especially in batteries, by suppressing dendrite formation and enhancing thermal stability. Consequently, ceramic electrolytes are ideal for use in liquid-free all-solid-state batteries (ASSBs).^17,18^

Several well-known FICs, such as Li_0.34_La_0.51_TiO_3_ (LLTO),^19^ Li_0.36_La_0.56_Ti_0.995_Al_0.005_O_3_ (Al-LLTO),^20^ Li_1.3_Al_0.3_Ti_1.7_(PO_4_)_3_ (LATP),^21^ and Na_3_Zr_2_Si_2_PO_12_ (NZSP),^22^ are frequently studied for ASSBs and could also have potential uses in SSCs. FICs typically do not develop a smooth interface, resulting in high interfacial resistance in addition to their inherent grain-boundary impedance (GBI).^23^ Thus, they have been combined with ionic liquids or ion-conducting polymers for SSC applications.

Recent reports on FICs-based SSCs are noteworthy. Various electrode-based supercapacitors were developed using solid electrolytes. These include ionic-liquid-incorporated LLTO, NZSP SSCs, and NZSP polymer composites.^24–26^

Among various FICs, Garnet-type Li^+^-ion conductor (Li^+^-garnets) are considered the best contender for energy storage, due to their much wider potential window, notably superior ionic conductivity, lower grain-boundary impedance, and most importantly, Li^+^-garnets are stable with Lithium metal anodes.^27^ The general formula for primary Li^+^-garnet is Li_7_A_3_B_2_O_12_, where A and B are cation sites occupied by 8- and 6-fold coordination.^28^ LLZO (Li_7_La_3_Zr_2_O_12_) is the most widely studied Li^+^-ion conducting garnet discovered by Ramaswamy Murugan and co-workers in 2007.^16^ It consists of an interconnected 3D framework of polyhedra, in which octahedra and tetrahedra are corner-sharing, forming a tunnel-like structure that facilitates the motion of Li^+^-ions. The chemical composition across the grain boundary and in the bulk of the grain is similar; thus, the formation of a space charge region is notably suppressed, resulting in very low grain boundary resistance compared to NASICONs and Perovskites and substantially high intragrain (bulk) conductivity (10^−3^–10^−4^ Ω^−1^ cm^−1^) at room temperature.^29^ The low electronic conductivity and strong ionic bonding widen the band gap, resulting in LLZO having the widest stable potential window, up to 6 V, with Li^+^/Li.^30,31^ This wide potential window enables high potential operation in pseudo-batteries and pseudo-capacitors. LLZO is known to exist in two phases: the tetragonal phase with space group *I*4_1_/*acd*, which has low ionic conductivity ∼10^−6^ Ω^−1^ cm^−1^ and the cubic phase with space group *Ia*3̄*d* which has a high ionic conductivity of ∼10^−4^ Ω^−1^ cm^−1^, but the cubic phase of LLZO is unstable at room temperature, thus aliovalent substitution (commonly Al^3+^, Ga^3+^, Y^3+^, Ta^5+^, Nb^5+^) is done to stabilize the cubic phase at room temperature by compensating the Zr site in pristine LLZO.^32^ The dopant not only stabilize the cubic phase but also enhances the ionic conductivity.^33,34^

Since its discovery, LLZO has attracted widespread attention, and over the past decade, Li^+^-garnet has been widely used in ASSBs.^35–38^ These batteries excel in energy density but lack power density. In an interesting work, for the first time, Kaur *et al.* synthesized an ionic liquid (∼6 wt%) LALZO (Li_6.75_Al_0.25_La_3_Zr_2_O_12_) composite for energy storage applications, delivering a capacity of ∼89 mA h g^−1^.^39^

The garnets somehow received limited attention for application in SSCs. This is possibly due to an interfacial contact issue, and the synthesis route for cubic garnet is complex and multistep. The mixed phase exhibits low ionic conductivity, inadequate for applications. Kaur *et al.* investigated the temperature tolerance of an ionic liquid (∼6 wt%) LALZO SSC from 0 to 100 °C. The device delivered a specific capacitance (*C*_S_) of ∼562 F g^−1^ at 2 V/1 mA, and exhibited stable GCD up to 4000 cycles, with a maximum specific energy of 86 Wh kg^−1^ (at 1.13 A g^−1^) and a specific power of 2195 W kg^−1^ (at 5.67 A g^−1^) at 35 °C.^40^

However, if the cubic phase of Li^+^-garnet is stabilized, its composite with an ion-conducting polymer can substantially improve the electrode–electrolyte interface without compromising the liquid-free characteristic of SSC. Therefore, this work has aimed to utilize the full potential of the cubic Li^+^-garnet composite polymer electrolyte in an electric double-layer capacitor (EDLC) geometry to explore its device applications.

This work has two important aspects: (i) to synthesize cubic-garnet based composite polymer electrolyte membrane of conductivity ≥10^−4^ Ω^−1^ cm^−1^, and assemble SSCs using this membrane, and (ii) enhancing the performance parameter by modifying the electrode–electrolyte interface. Herein, the cubic phase of niobium (Nb)- doped garnet with a stoichiometric formula of Li_6.75_La_3_Zr_1.75_Nb_0.25_O_12_ (LLZNO) with excess ∼15 wt% lithium salt is reported.^41^ The optimized composition of Li^+^-garnet hybrid composite polymer electrolyte (LG-HCPE) has been reported as 12LiClO_4_ + 88{(PEO)_0.55_ (LLZNO)_0.45_}. The optimized composition is then utilized to fabricate SSCs with AC electrodes (surface area ∼1800 m^2^ g^−1^) in a symmetric geometry. It has been observed that incorporating an organic solvent, such as DMF, onto the electrolyte surface forms a micro-gel layer that facilitates interfacial ion transport, leading to high SSC performance near, and in fact, below room temperature. Furthermore, the SSC exhibits stable performance up to 3 V, with a minimal IR drop and high coulombic efficiency. In fact, these SSCs are stable up to 100 000 GCD cycles, with reasonably high specific capacitance retention. These results stand out for cubic Li^+^-garnet among all other ceramic electrolytes.

## 2 Experimental: materials and methods

### 2.1 Materials

High-purity materials for synthesizing Li^+^-ion garnet and its composite with PEO were procured, *viz.* Lithium hydroxide monohydrate (LiOH.H_2_O: Thermo Scientific ACS 99%), lanthanum(iii) oxide (La_2_O_3_: Thermo Scientific 99.99%), zirconium dioxide (ZrO_2_: Molychem 99.5%), niobium pentoxide (Nb_2_O_5_: CDH 99.5%), lithium perchlorate (LiClO_4_: Thermo scientific 99%), polyethylene oxide (PEO: Alfa Aesar MW ∼300 000 g mol^−1^), activated carbon (AC: ASG scientific, surface area ∼1800 m^2^ g^−1^), poly(vinylidene fluoride-*co*-hexafluoropropylene) (PVDF-HFP: Sigma-Aldrich, MW ∼400 000 g mol^−1^), and acetylene black (AB: Alfa Aesar 99.9+% surface area 75 m^2^ g^−1^), *N*,*N*-dimethylformamide (DMF: RANKEM 99%). These materials were used without additional purifications.

### 2.2 Synthesis: Li^+^-ion garnet-type LLZNO

**Fig. 1:**
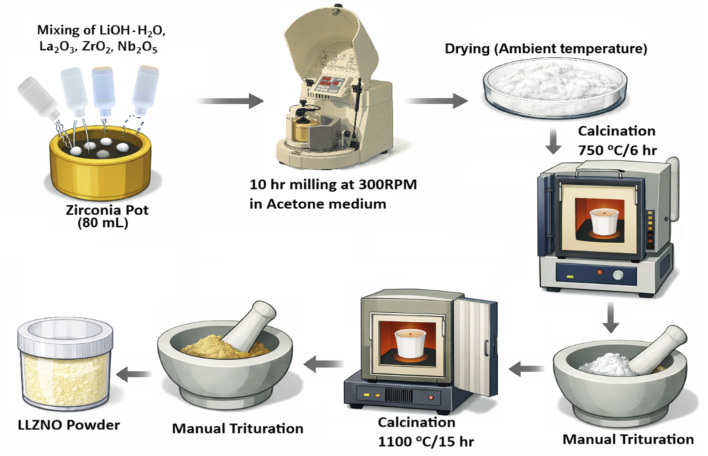
Synthesis route of LLZNO garnet *via* the solid-state reaction.

The lithium garnet with a nominal composition Li_6.75_La_3_Zr_1.75_Nb_0.25_O_12_ (LLZNO: lithium lanthanum zirconium niobium oxide) was prepared *via* the conventional solid-state reaction route as in Fig. 1, where the precursor contains the stoichiometric amount of LiOH·H_2_O, La_2_O_3_, ZrO_2_ and Nb_2_O_5_. With an excess of 15 wt% of LiOH·H_2_O to compensate for the lithium loss during the high-temperature treatment. La_2_O_3_ was pre-dried at 900 °C for 12 h.^42–44^ The precursor powder was then ball-milled in a zirconia pot using a planetary ball mill (Fritsch-P6) in acetone medium for 10 h at 300 rpm. After thorough mixing of the precursor compounds, the solvent was allowed to evaporate at ambient temperature, and then heated in a high-purity alumina crucible to 750 °C for 6 h. The mixture was cooled and ground into a fine powder. Further, the fine powder was calcined at 1100 °C for 15 h at a heating rate of 5 °C min^−1^, then again cooled to room temperature under normal conditions. Finally, the sample is pulverised to obtain a fine powder of Li^+^-garnet.

### 2.3 Synthesis: Li^+^-ion garnet hybrid composite polymer electrolytes (LG-HCPEs)

**Fig. 2:**
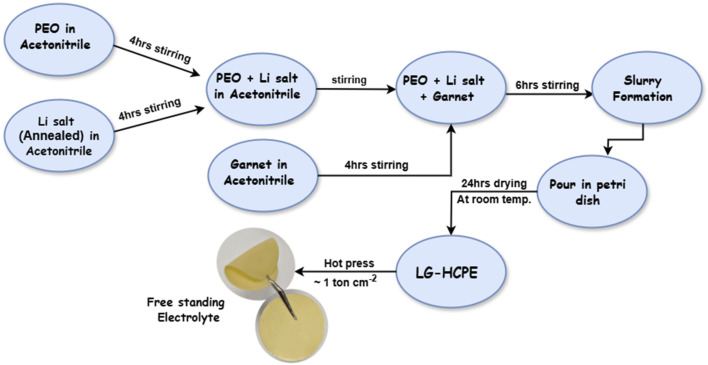
The preparation route of LG-HCPE. The original picture of the free-standing membrane is shown. The composite membrane is flexible and does not require any support to maintain its shape.

The hybrid composite polymer electrolyte infused with cubic Li^+^-garnet was prepared using the traditional solution casting (Fig. 2). First, the salt (LiClO_4_) was pre-annealed at 150 °C for 2 hours. The polymer host matrix (PEO) and the prepared Li^+^-garnet were separately dissolved in acetonitrile and stirred for 4 h without heating until a uniform solution was obtained. Furthermore, all three solutions were blended and stirred at 400 rpm for 6 h, until a homogeneous slurry formed, which was then uniformly poured onto the Teflon Petri dish and allowed to dry for 24 h at ambient temperature and pressure. The general compositional formula used for the synthesis of LG-HCPE in the present work is *x*LiClO_4_ + (100 − *x*) {(PEO)_1−*y*_ (LLZNO)_*y*_}, where *x* varied between 5–15 wt%, and *y* varied between 0–0.8. Here, LLZNO substitutes PEO in the matrix. For example, 45LLZNO_12S is designated for a composition containing 12 wt% of salt and 45% of LLZNO (39.6 in wt%) and 48.4 wt% PEO in the remaining matrix. Hereafter, the electrolyte configuration is abbreviated as yLLZNO_12S, where ‘*y*’ is the LLZNO fraction substituting PEO in the matrix. Such a substitution reduces the PEO content in the matrix, while keeping the fixed salt amount, thereby altering the O/Li ratio in the LG-HCPE.

Finally, the dried slurry was hot-pressed using a polymer press at ∼1 ton cm^−2^ to form a Li^+^-ion garnet hybrid composite polymer electrolyte membrane of thickness ∼200 µm for further characterization.

### 2.4 Supercapacitor fabrication

**Fig. 3:**
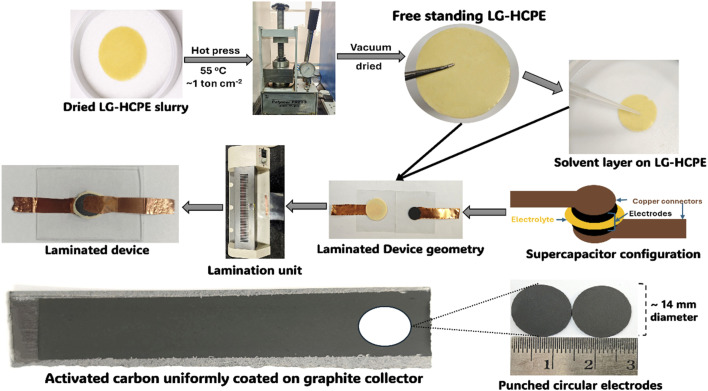
Fabrication process for LG-HCPE infused SSCs in laminated device geometry.

The Prepared LG-HCPEs were assembled in a symmetric supercapacitor configuration (Cu|AC|LG-HCPE|AC|Cu) using AC electrodes on both sides of the electrolyte. The electrode material was prepared using activated carbon, PVDF-HFP binder, and acetylene black as a conducting agent, which was dissolved in *N*-methyl-2-pyrolidone (NMP) in an 80 : 10 : 10 ratio and stirred until a uniform slurry was formed. The slurry was then placed in a vacuum mixer (AOTELEC: AOT-AX-2000) to remove trapped air, then coated onto a ∼0.45 mm thick graphite sheet using a desktop coating machine (Aero: AC 250), and vacuum-dried at 60 °C for 24 h. Subsequently, the electrode was passed through a calendaring machine (AOTELEC: AOT-SG-100V) to ensure a uniform thickness. The active mass loading on the graphite strip is maintained at ∼1.1 mg cm^−2^. The strip was then punched into a circular electrode with a diameter of 14 mm (area ∼1.54 cm^2^) and used to assemble the SSCs. The synthesized LG-HCPE was then sandwiched between the punched graphite electrodes along with copper connectors and passed through a hot-roll laminator to obtain SSC in a laminated geometry (Fig. 3).

To improve the interface between the electrodes and the electrolyte in SSCs, a micro-gel layer was formed on the membrane surface using a minimal amount (∼5 µL cm^−2^) of DMF (as elaborated later in 3.6.2).

The assembled SSCs are then used for further electrochemical characterizations.

#### 2.4.1 Electrochemical parameters assessment

To study the electrochemical performance parameters of the SSC, the following formulas have been used.^45–49^

(i) Coulombic efficiency: 1
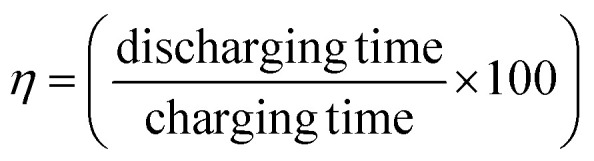


(ii) Total capacitance from CV:2
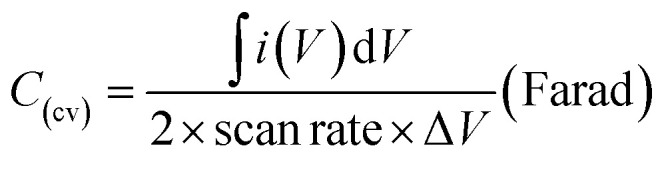
where ∫*i*(*v*)d*v* is the area under the CV curve, scan rate (V s^−1^), and Δ*V* is the working potential window (*V*_2_–*V*_1_) in the CV plots.

(iii) Specific capacitance from CV: 3
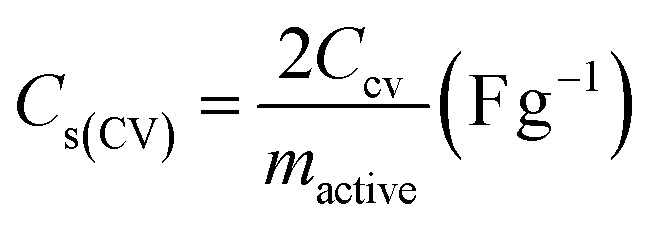
where *C*_cv_ is the capacitance calculated from the CV curve, and *m*_active_ (g) is the average mass of the active material deposited on a single electrode.

(iv) Total capacitance from GCD: 4
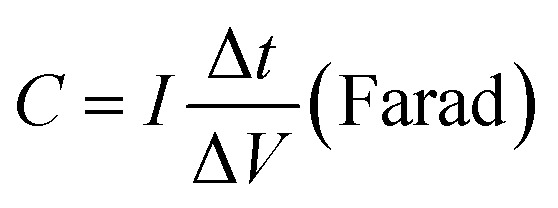
where *I* (A) is the discharging current, Δ*t* (sec) is the discharging time, and Δ*V*(v) is the initial voltage for the discharging cycle (excluding IR drop).(Δ*V* = *V*_max_ − *V*_IR_)

(v) Specific Capacitance from GCD: 5
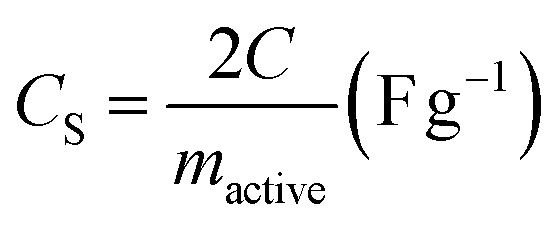
where *C* is in Farad (F), and *m*_active_ is the average mass (g) of the active material (*i.e.* AC) on a single electrode.

(vi) Specific energy (full device): 6
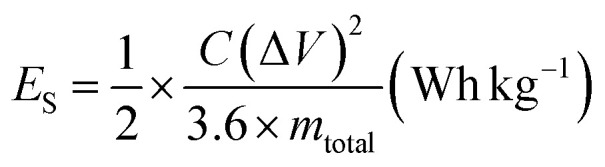
where *m*_total_ is the total mass of active material on both the carbon electrodes (*m*_total_ = *m*_1active_ + *m*_2active_).

(vii) Specific power (full device): 7
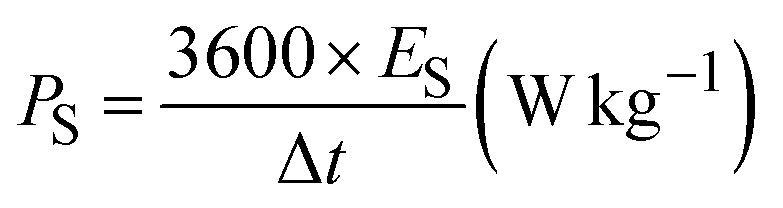


(viii) Equivalent series resistance (from GCD): 8
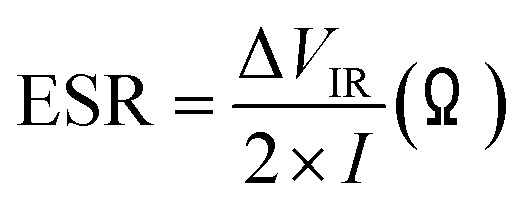
where Δ*V*_IR_ (V) is the initial voltage drop in the discharging cycle, and *I* (A) is the discharging current. The normalized ESR (Ω cm^2^) is calculated by multiplying ESR by the electrode area (∼1.54 cm^2^).

### 2.5 Characterization

The synthesized LLZNO garnet and LG-HCPE were characterized using different structural, morphological, thermal, chemical and electrochemical techniques as mentioned below.

X-ray diffraction (XRD) measurements of pristine Li^+^-garnet and its polymer composites were performed using a Rigaku Miniflex II X-ray diffractometer with Cu-K_α_ radiation of *λ* = 1.54 Å with the scan rate of 1° min^−1^. The obtained XRD spectra were used for Rietveld refinement using FullProf software to determine the lattice, atomic, and shape parameters. The crystal structure was visualized using the VESTA software. Morphological and microstructural analysis of LLZNO particles and LG-HCPE were investigated using Field Emission Scanning Electron Microscopy (FESEM: FEI-Apreo-LoVac). Phase transition, degree of crystallinity, and melting temperatures of LG-HCPE were analyzed using Differential Scanning Calorimetry (DSC: Shimadzu DSC-60) from room temperature to 100 °C, while the thermal stability of the LG-HCPE with variable LLZNO content was studied using Thermogravimetric Analysis (TGA: Shimadzu TGA-50) from room temperature to 500 °C. Both at a heating rate of 10 °C min^−1^ under an inert (Nitrogen) atmosphere. Chemical bonding, molecular vibrations and interactions were observed using Fourier Transform Infrared Spectroscopy (FTIR: Shimadzu IR Spirit-T), and Raman spectroscopy (HORIBA, AIST-NT Labram HR Evolution, Omega Scope). The contact angle measurement was performed on a Kruss Gmbh DSA25B contact angle meter at room temperature.

The steady state electrical transport measurements of LG-HCPE were performed in a wide temperature range (−70 to 100 °C) and in a frequency range 4 Hz–5 MHz using an HIOKI IM3570 impedance analyzer. For the ionic transference number measurement, DC current is measured using a KEYSIGHT 34461A Digital Multimeter. A homemade cryostat was used for measurements below room temperature.

Finally, the device performance of assembled SSCs, including Electrochemical Impedance Spectroscopy (EIS), Bode plot analysis, Cyclic Voltammetry (CV), Linear Sweep Voltammetry (LSV), and galvanostatic charge–discharge (GCD), was measured using a Metrohm Autolab 204 electrochemical workstation. Further, prolonged cycling of SSCs was performed using a NEWARE battery tester (MHW-25).

## 3 Results and discussion

### 3.1 X-ray diffraction (XRD)

**Fig. 4:**
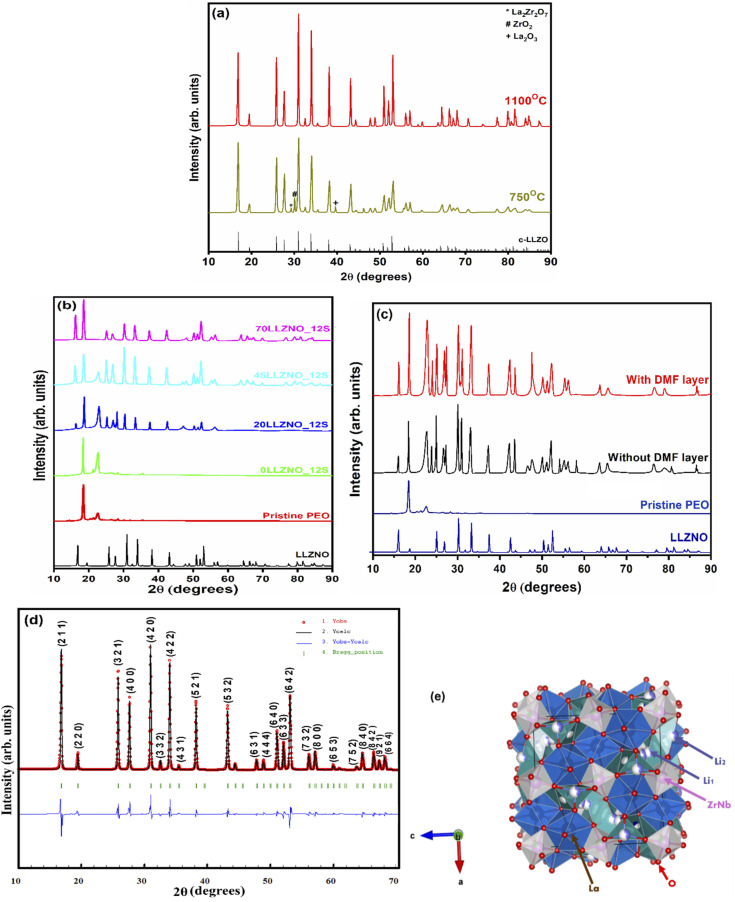
XRD spectra: (a) synthesized LLZNO powder at 750 and 1100 °C, (b) Composite of xLLZNO_12S, (c) LG-HCPE with and without incorporation of DMF layer, (d) Rietveld refinement of synthesized LLZNO, (e) crystal structure of LLZNO from VESTA.

The structural analysis of pristine LLZNO and its composite electrolytes has been studied using X-ray diffraction. The powder XRD of pristine LLZNO was performed after each calcination process (*i.e.* 750 °C and 1100 °C) as shown in Fig. 4(a). The formation of cubic garnet (ICSD:00-064-0141) is witnessed at 750 °C, with minor impurity peaks (corresponding to ZrO_2_, La_2_O_3_, and La_2_Zr_2_O_7_) present in small amounts, possibly due to incomplete reaction.^50–53^ After calcination at 1100 °C, the impurity peaks disappear, and a pure cubic LLZNO phase is confirmed. Here, diffusion facilitates Zr incorporation into the garnet lattice.^54,55^ Also, after high-temperature treatment, the peaks appear sharper, indicating improved crystallinity and larger crystallites. The average crystallite size of LLZNO garnet is ∼50 nm, determined using the Debye–Scherrer formula.

Fig. 4(b) shows the XRD pattern of LG-HCPE at different concentrations of LLZNO garnet. Initial addition of salt in the PEO matrix reduces the crystallinity of the PEO, which is evident by the suppressed peak of crystalline PEO.^56^ As the LLZNO concentration increases, the PEO volume fraction in the composite electrolyte decreases, and prominent LLZNO peaks appear for 70LLZNO_12S (having 61.6 wt% of LLZNO). The LLZNO intensities in the composite polymer electrolyte are low. This could be because the LLZNO particles are well distributed and embedded deep within the polymer matrix. Their concentration is also low (39.6 wt%), resulting in fewer crystallites being illuminated and contributing less to the intensity. As the LLZNO concentration increases in the matrix, the LLZNO peak intensities are relatively higher, indicative of a dominant LLZNO contribution. Furthermore, no additional peaks were observed during composite formation, indicating that no new crystalline phase forms, and LLZNO particles remain in a separate phase embedded in the polymer matrix.

Fig. 4(c) shows the XRD spectra of LG-HCPE with and without the incorporation of DMF onto the membrane surface. The spectra indicate that no new crystalline compound or phase forms upon the introduction of the DMF layer.^57^ Also, the peak positions remain almost unchanged, suggesting no change in the planar spacing or other cell parameters of LLZNO. Though the peak intensity of LLZNO improves notably. It suggests that adding DMF improves exposure of ceramic crystallites to the X-rays by thinning/softening the polymer layer and reducing polymer scattering, thereby allowing more crystal planes to contribute to scattering.

Fig. 4(d) represents the Rietveld refinement analysis of the powder LLZNO at room temperature with reference to the crystallographic information file (reference number 1556981) obtained from the crystallographic open database (COD). After refinement, the XRD spectra of powder LLZNO are well aligned with the reference cubic LLZNO data, exhibiting excellent fitting with the goodness of fit (*χ*^2^) ∼1.43. The fitting also confirms that no phase separation occurs after incorporating niobium into the LLZO matrix. The refinement also confirms the formation of the cubic phase with *Ia*3̄*d* space group of the obtained LLZNO at room temperature.^58^ The refinement yields cell parameters *a* = *b* = *c* = 12.899 Å and *α* = *β* = *γ* = 90°, with unit cell volume of ∼2146.189 Å^3^.

The corner-sharing polyhedra structure is obtained using VESTA, based on the Rietveld refinement parameters (Fig. 4(e)). As is evident, the LiO_4_ tetrahedra (Li_1_) and LiO_6_ distorted-octahedra (Li_2_) share corners forming a 3D interconnected tunnel-like structure, which eventually facilitates the motion of Li^+^-ion in the matrix and thereby enhances the Li^+^-ion transport in LLZNO garnets, as reported previously.^59,60^

### 3.2 Surface morphology and microstructure analysis

FESEM is used to investigate the morphology and microstructure of LLZNO and its polymer composites.

**Fig. 5:**
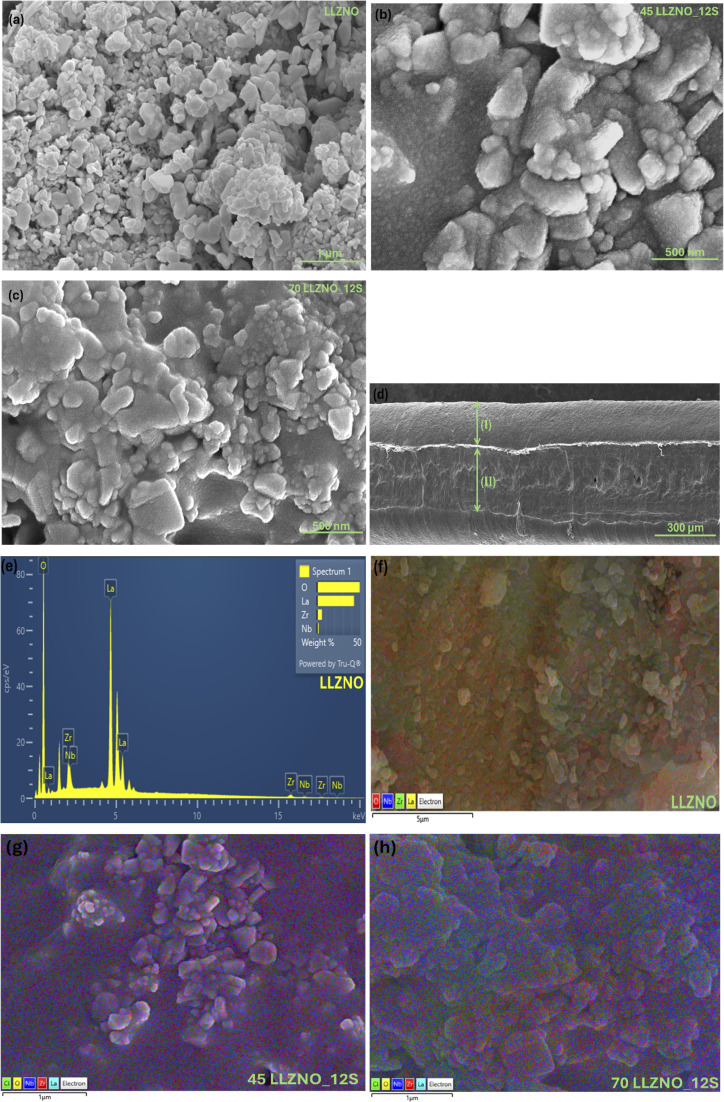
FESEM images: (a) Pristine LLZNO, (b) and (c) 45LLZNO_12S and 70LLZNO_12S, (d) micro-gel layer on LG-HCPE after DMF incorporation, (e) elemental distribution spectrum of LLZNO, (f)–(h) elemental mapping of pristine LLZNO, 45LLZNO_12S, and 70LLZNO_12S samples.

Fig. 5(a) shows the micrograph of pristine LLZNO powder, which reveals a uniform morphology of LLZNO powder. The grains are almost of uniform size. Fig. 5(b) and (c) show the images of the polymer electrolyte with LLZNO crystallites. A homogeneous distribution of LLZNO is seen in the polymer matrix (Fig. (5b)). When the LLZNO content is increased in the matrix, *i.e.* 70LLZNO_12S, one observes a clear interconnected network of grains, with polymer bridging the gaps (Fig. 5(c)). This facilitates easy ion migration, thereby increasing ion mobility and ionic conductivity.

Fig. 5(d) shows a cross-sectional view of micro-gel layer formation when DMF is added onto the surface of LG-HCPE. This thin DMF-diffused gel layer appears to be of almost uniform thickness of ∼50–80 µm (region I) over the surface of the polymer electrolyte of thickness ∼200 µm (region II).

Fig. 5(e) shows the elemental distribution of LLZNO, clearly indicating the presence of all precursor elements (except Li, which emits a very small characteristic X-ray at ∼0.055 keV, which is far lower than the detection limit) with negligible impurities. The elemental mapping of LLZNO in Fig. 5(f), composite electrolyte with 45LLZNO_12S in Fig. 5(g), and 70LLZNO _12S in Fig. 5(h), shows a homogeneous distribution of all elements across the surfaces of LLZNO and its composite electrolytes.

### 3.3 Physicochemical analysis

To understand the physicochemical properties, chemical bonding, molecular structure, and the interactions between several groups, FTIR and Raman studies of LG-HCPE were conducted.

**Fig. 6:**
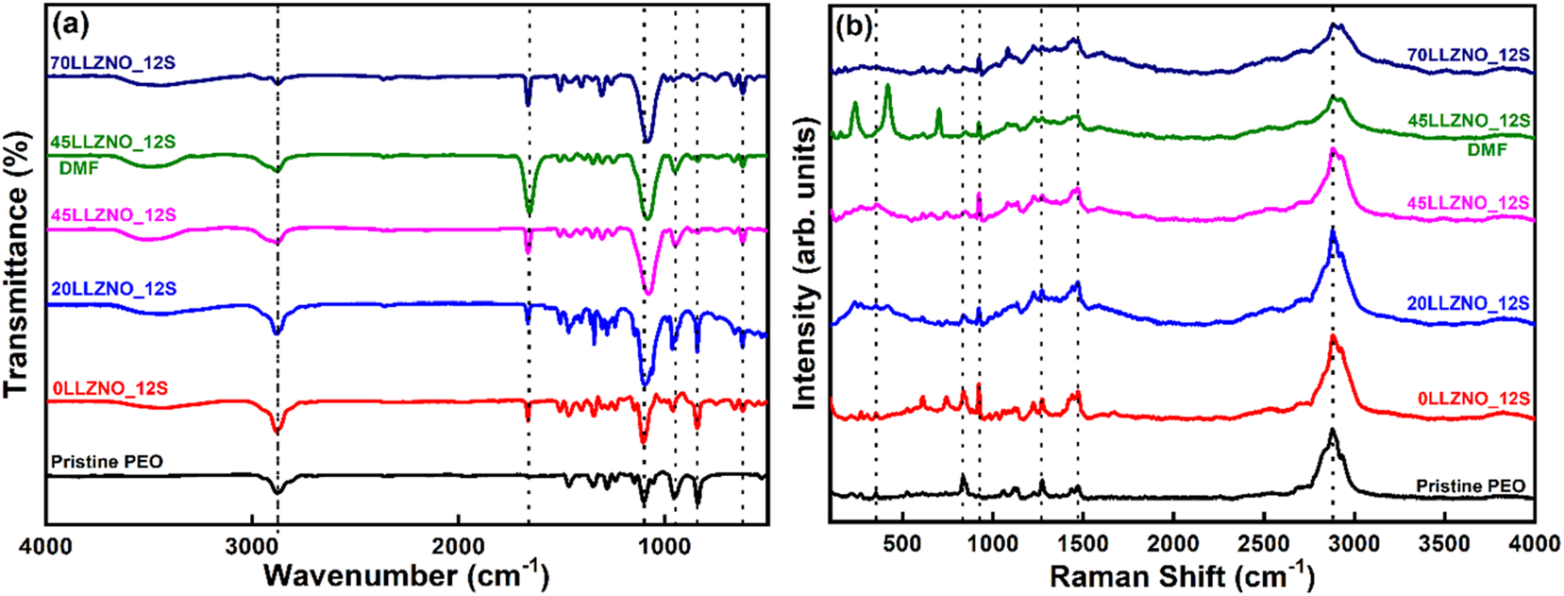
(a) FTIR (b) Raman profiles for PEO and its composite electrolyte along with DMF insertion.

Fig. 6(a) shows the FTIR spectra of PEO, PEO + LiClO_4_, and the composite polymer electrolyte with different LLZNO concentrations in the region of 500 to 4000 cm^−1^. The fingerprint region (400–1500 cm^−1^) shows several peaks, out of which some weak peaks (500–800 cm^−1^) are due to skeletal polymer backbone vibration of –(CH_2_–CH_2_–O)– and CH_2_ deformation modes. The sharp peak at ∼840 cm^−1^ confirms the crystalline phase of pristine PEO. Peaks at ∼956, 1280, and 1466 cm^−1^ may be assigned to CH_2_ rocking, twisting, and bending vibrations. Further, the peak at ∼1100–1110 cm^−1^ confirms the C–O–C symmetric–asymmetric ether vibration that is the backbone of the PEO, and the peak at ∼2880 cm^−1^ corresponds to the symmetric–asymmetric stretching of CH_2_.^61^ The characteristic peak of LiClO_4_ appears at ∼615, 1100, and 1635 cm^−1^, indicating bending and stretching of the free ClO_4_^−^ ion. The single sharp peak at ∼1100 cm^−1^ suggests the presence of free Li^+^ ions and no ion agglomeration/ion pairing.^62^ Peaks between ∼620–700 cm^−1^ correspond to Zr–O stretching and bending, while ∼3200–3600 cm^−1^ corresponds to O–H stretching, due to the hygroscopic nature of LLZNO and LiClO_4_. It is observed that upon addition of LiClO_4_ and LLZNO, the crystalline peak area of PEO at ∼840 and 956 cm^−1^ decreases significantly, suggesting an increase in the amorphous phase. Also, the area under several other peaks at ∼956, 1280, and 1466 cm^−1^ decreases with the addition of LLZNO, confirming a decrease in the polymer phase. The sharp peak at ∼1100 cm^−1^ broadens and shifts toward lower wavenumbers. This indicates less ordering and more Li^+^-ion coordination with the ether oxygen (–O–) in the polymer chain, and an increase in the amorphous phase in LG-HCPE, which facilitates ion transport (discussed later in Section 3.4). No new phase forms upon addition of LLZNO to the polymer matrix. A sharp peak at ∼1660–1670 cm^−1^ arises due to the stretching of the carbonyl group (C=O) of DMF.^63^ Upon the incorporation of DMF onto the LG-HCPE surface, again, no new absorption peaks appear in the spectrum.

Raman spectra (as in Fig. 6(b)) also probe the functional groups and chemical bonds in PEO, PEO + LiClO_4_, and LG-HCPE. The characteristic peaks of PEO appear at ∼838, 1278, and 1420–1490 cm^−1^, arising from CH_2_ deformation (rocking, twisting, and bending). The peaks at ∼2880 cm^−1^ appear due to the symmetric and asymmetric stretching of CH_2_, and the peaks at ∼1058 cm^−1^ show C–C stretching, while the peak at ∼1130 cm^−1^ shows the stretching vibration of ether oxygen in C–O–C.^64^ Peaks at ∼610, and 1110–1150 cm^−1^ suggest bending and asymmetric stretching of ClO_4_^−^ ion. The sharp peak at ∼925 cm^−1^ indicates strong symmetric stretching of the free ClO_4_^−^ ion. Upon the insertion of LiClO_4_ and LLZNO, there is a significant decrease in the intensity of PEO peaks (∼836, 1273, and 1472 cm^−1^), the peak at ∼2880 cm^−1^ broadens and is suppressed with increasing LLZNO content, suggesting a significant increase in the amorphous phase in the composite electrolyte. Peaks at ∼220–240, 400–420, and 670–700 cm^−1^ arise from the addition of DMF and correspond to the bending of O=C–N.^65–67^ No further bonding or phase formation is observed upon addition of DMF to LG-HCPE.

The above discussion on FTIR and Raman analysis complements each other and concludes that (i) PEO gradually attains more disorder when LLZNO is reinforced into the matrix, (ii) there is no direct evidence of any strong complexation of PEO and LLZNO, and (iii) DMF addition broadens the peaks of PEO, indicative of micro-gel formation on the surface of the polymer electrolyte as evident from FESEM.

### 3.4 Thermal analysis

**Fig. 7:**
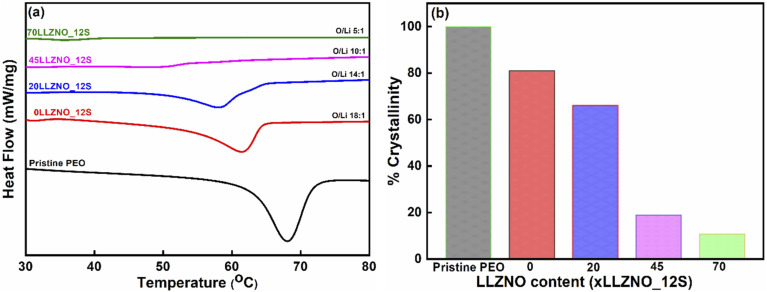
(a) DSC thermograph of PEO and its composites, (b) variation of crystallinity of PEO in the composites with LLZNO content.

To understand the thermal behaviour of LG-HCPE, Differential scanning calorimetry (DSC) was performed. Fig. 7(a) shows the DSC thermogram of pristine PEO, PEO + 12LiClO_4_, and LLZNO variation in the composite electrolyte. The heat flow is normalized with the actual mass of the PEO in the composite. Evidently, an endothermic peak (onset around 60 °C) corresponds to the melting of pristine PEO.^68^ With the further addition of salt and LLZNO, the area under the peak decreases gradually, and the peak also shifts towards lower temperatures. This implies that the addition of salt and LLZNO disrupts the crystalline structure of PEO. Apparently, it requires less thermal energy to melt. The amorphous phase of PEO in the composite electrolyte thus increases, though its amount also decreases due to the substitution of LLZNO. For 45LLZNO_12S, the peak area reduces substantially, and for 70LLZNO_12S, the peak almost disappears, suggesting that the composite electrolyte is almost entirely in the amorphous phase. This promotes ion mobility in the electrolyte, thereby enhancing the overall conductivity of LG-HCPE.

The area under the peak is proportional to the enthalpy change (Δ*H*). Fig. 7(b) shows the change in the crystallinity of the polymer relative to the PEO, which is calculated using the relation:9
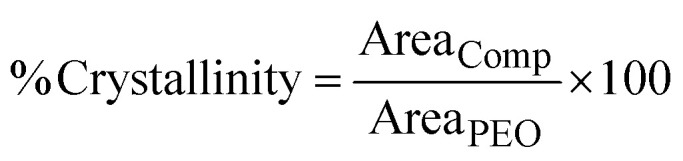
where Area_comp_ is the area under the curve for the composite polymer electrolyte, and Area_PEO_ corresponds to that of pristine PEO.

It may be emphasized here that, when LiClO_4_ is first added to the polymer, its crystallinity reduces substantially. When salt concentration is fixed to 12 wt%, and PEO is substituted by LLZNO, the crystallinity of the composite electrolyte further decreases significantly. The addition of LLZNO further amorphizes the PEO, increasing segmental motion, and a visible change in crystallinity is observed. Similar observations have been reported by Singh *et al.*, where the % crystallinity shows a similar trend with LTP incorporation into the PEO-LiCF_3_SO_3_ matrix.^69^ The addition of LLZNO to the composite electrolytes also improves the resistance to decomposition (∼360 °C), as evident in the thermogravimetric analysis (TGA) (SI Fig. S1).

### 3.5 Electrical transport in LG-HCPE membranes

**Fig. 8:**
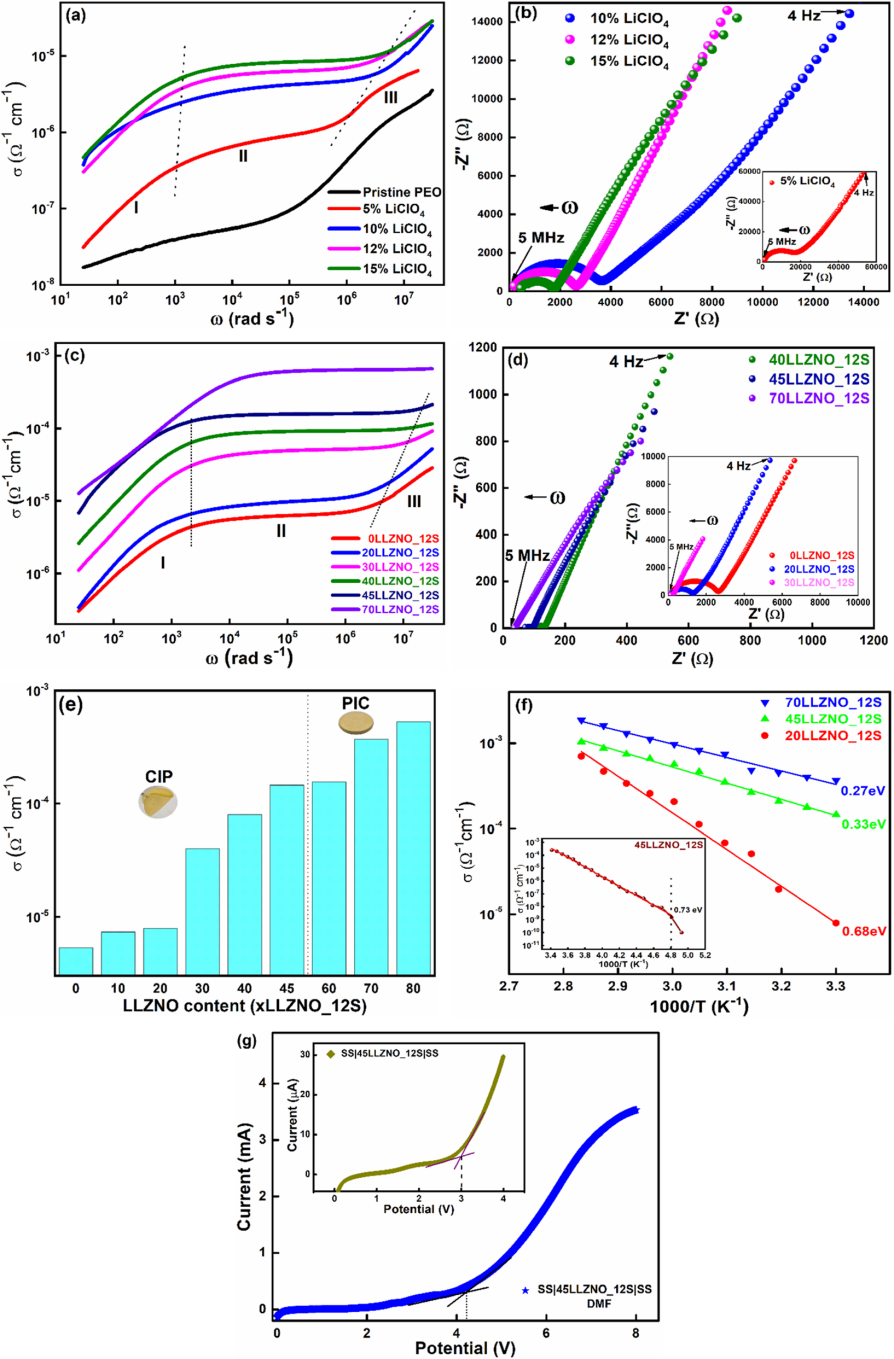
(a) Conductivity variation with LiClO_4_ concentration, (b) Nyquist plot with variable LiClO_4_, inset shows the Nyquist plot 5% LiClO_4_, (c) angular frequency (*ω*) dependent conductivity with variable LLZNO, (d) Nyquist plot for 40, 45, and 70LLZNO_12S, inset shows the Nyquist plot at 0, 20, and 30LLZNO_12S, (e) conductivity variation with LLZNO content, (f) temperature dependence of the conductivity, inset shows the behaviour of conductivity at low temperatures, (g) LSV of LG-HCPE with DMF, inset shows LSV of LG-HCPE without DMF.

Electrical transport in the composite electrolyte (LG-HCPE) was studied over a wide temperature range. At the outset, the electrical transport in PEO + LiClO_4_ membranes was investigated. Fig. 8(a) shows the frequency dependence of conductivity at varying LiClO_4_ concentrations. In the low-frequency range (Region I), the conductivity drops rapidly with decreasing frequency. This is because ions have sufficient time to migrate and accumulate at the electrode–electrolyte interface, leading to interfacial polarization, which is first-hand evidence of predominant ionic transport. At intermediate frequency (region II), conductivity exhibits a plateau, often referred to as the DC conductivity region, where it remains at a nearly constant value. In region III, the dispersion region (frequency ≥1 MHz), ions lack long-range diffusive motion, resulting in localized hopping and causing dispersion in conductivity.^70^ The salt concentration is optimized at 12 wt%, as higher content leads to salt precipitation and causes the composite to absorb moisture. The conductivity of a pristine PEO (without salt and ceramic fillers) lies in the range of ∼10^−8^ Ω^−1^ cm^−1^ at room temperature. With an increase in salt content from 0 to 15 wt%, the conductivity increases considerably, reaching ∼10^−6^ Ω^−1^ cm^−1^ at 30 °C.

The semicircular feature (Fig. 8(b)) in the high-frequency region arises from the bulk of the polymer electrolyte, with contributions from ionic motion and segmental relaxation. The depressed semicircle refers to the distribution of relaxation times, and the diameter refers to bulk resistance, while the low-frequency tail is due to electrode polarization. As salt content increases (5 to 15 wt%), the diameter of the semicircle decreases considerably compared to 5 wt% salt (inset) and shifts toward higher frequencies. Salt dissolves into the polymer matrix, introducing mobile ions that enhance ion transport.

Fig. 8(c) shows the frequency dependence of the conductivity (*σ*–*ω* plot, 30 °C) for the composite reinforced with different LLZNO concentrations. The conductivity increases with increasing LLZNO content in the matrix. Fig. 8(d) shows the Nyquist plot for the variation of LLZNO content in LG-HCPE. This is reflected in the Nyquist plot, where the semicircle diameter further decreases with increasing LLZNO. The semicircle shifts away from the origin, suggesting the role of LLZNO in promoting electrical transport. The rise in the conductivity may be due to (i) amorphization/disorder created in the polymer (PEO), as also suggested by DSC (Fig. 7), or (ii) contribution of LLZNO grains in facilitating electrical transport *via* polymer-LLZNO interface.^71^

Fig. 8(e) shows the conductivity variation with the LLZNO content in both “ceramic-in-polymer (CIP), where *y* ≤ 0.45 and “polymer-in-ceramic (PIC), *y* > 0.45 configurations. In PICs, LLZNO occupies a major volume in the matrix, and forming a thin membrane is not feasible. Therefore, the measurements were conducted on a thin pellet/sheet of the composite. Conductivity shows a gradual monotonic rise with LLZNO content, though the mechanism of conductivity is likely to be different in PIC and CIP.^72,73^

Fig. 8(f) shows the temperature-dependent conductivity of LG-HCPE with different LLZNO compositions. For the optimized configuration (45LLZNO_12S), the ionic conductivity (*σ*_DC_) is found to be ∼2 × 10^−4^ Ω^−1^ cm^−1^ at 30 °C, which is four orders of magnitude higher compared to pristine PEO. As temperature increases, polymer (PEO) chains in the electrolyte gain thermal energy and become more flexible, leading to increased segmental motion and easier ion hopping. This leads to an increase in conductivity. In a bare PEO + LiClO_4_, the mobile Li^+^-ions couple their motion with the ether oxygen of the PEO, and this ion-chain coupling is responsible for electrical transport. The conductivity of garnet-polymer composites rises Arrheniusly, as also observed in other polymer-ceramic composites.^72,74^ This is apparent when the conductivity is measured over a wide temperature range. Such linear behaviour indicates decoupling of mobile ions from the polymer chains and may be attributed to the presence of LLZNO in the matrix.^71^

The inset of Fig. 8(f) shows the low-temperature electrical transport in 45LLZNO_12S, ranging from 20 to −70 °C. Conductivity rise is almost Arrhenius, and a linear temperature dependence is observed till −60 °C. (i) VTF behaviour, which is commonly witnessed in polymer-salt composites, is not observed in this case.^75^ This suggests a possibility of decoupling of mobile ions from the polymer chains. These decoupled ions contribute more significantly to electrical transport. (ii) Up to a temperature of −10 °C, the LG-HCPE show conductivity in the order of ∼10^−5^ Ω^−1^ cm^−1^ adequate to electrolytic applications. (iii) Further reducing the temperature ≤*T*_g_ of the PEO (around −60 to −65 °C), the conductivity falls rapidly below −65 °C (marked by dotted line). Below *T*_g_, the localized chain motion and flexing freeze, causing the polymer to become rigid and dense.^76^ As a result, thermal activation for ionic transport is inadequate. This restricts ion transport, leading to a large drop in conductivity.

The total conductivity of the composite is predominantly ionic, as evident by the ionic transference number ∼0.998 (close to unity), measured by the symmetric Hebb–Wagner DC polarization method (SI Fig. S2).

Fig. 8(g) shows Linear sweep voltammetry (LSV) of LG-HCPE performed at a scan rate of 10 mV s^−1^ under ambient conditions, in the blocking stainless steel electrode (SS) configuration. The electrolyte membrane remains stable up to ∼3 V (inset). The stability window extends to ∼4 V after adding DMF to the electrolyte surface, most likely due to reduced polarization effects. For safer device application, the potential window is capped at 2 V.

### 3.6 LG-HCPE supercapacitor: electrochemical performance

The LG-HCPE with the optimized configuration 45LLZNO_12S is then assembled into an SSC geometry, with AC electrodes sandwiching the solid electrolyte membrane, and the electrochemical properties, including EIS, CV, GCD, and long cycling were tested. Although 80LLZNO_12S exhibits the highest conductivity, it was avoided for SSC applications because of the inability to form a thin membrane due to high ceramic content, which may lead to poor interfacial contacts.

#### 3.6.1 Electrochemical performance of the bare device (without interface alteration)

It was considered important to understand how the untreated solid electrode–electrolyte membrane interface behaves under supercapacitor conditions. Therefore, the first set of devices was assembled with a bare, unaltered interface.

**Fig. 9:**
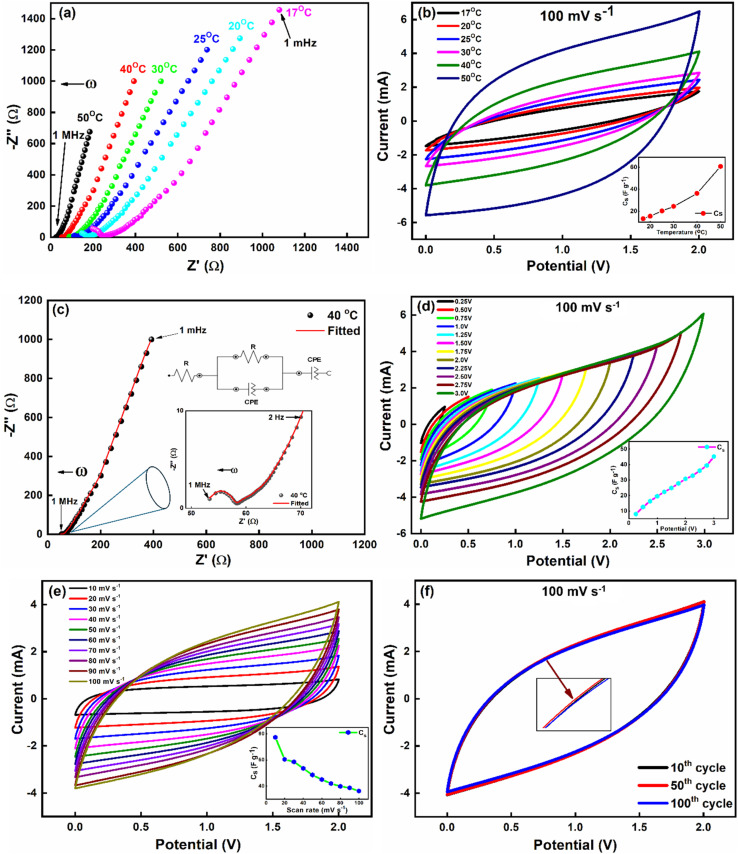
(a) Nyquist plots of bare device at different temperatures, (b) CV profile at different temperatures, inset shows *C*_S_ (from CV) as a function of temperature, (c) Nyquist plot of the SSC at 40 °C, inset shows the equivalent circuit and extended view of high frequency region, (d) CV profile corresponding to different potential windows at 100 mV s^−1^, inset shows *C*_S_ (from CV) with potential variation, (e) CV at different scan rates, inset shows *C*_S_ (from CV) variation with scan rate, (f) first 100 CV cycles at 2 V/100 mV s^−1^, inset shows zoom view of cycles.

Fig. 9(a) presents the Nyquist plots of the SSC at different temperatures ranging from 17 to 50 °C, over a wide frequency range of 1 mHz to 1 MHz. Interestingly, when the temperature increases, the polymer electrolyte membrane conductivity also increases, enabling ions to rapidly reach the interface and cover most of the surface area of the AC electrode. High electrolyte conductivity also improves interfacial diffusion, thereby reducing the IR drop as well as the ESR. As apparent in Fig. 9(a), the diameter of the semicircle (full SSC device) decreases with increasing temperature. The ESR gradually decreases from ∼382 Ω cm^2^ (17 °C) to ∼38 Ω cm^2^ (50 °C).

Fig. 9(b) presents the CV profile for a potential window of 2 V/100 mV s^−1^ scan rate at different temperatures. As the temperature increases, the area under the CV plots increases gradually, and the corresponding current increases. This might be due to fast charge-transfer kinetics, improved contact between the electrode and the electrolyte, and increased conductivity, leading to higher current in CV, eventually, an increase in the area under the curve, and a more rectangular shape.^77^ The inset shows the gradual increase in *C*_S_ with increasing temperature.

Fig. 9(c) shows the Nyquist plot of the device at 40 °C, along with its equivalent circuit. The same, on an extended scale, is shown in the inset. The plot shows the device impedance profile, featuring a tiny semicircle at a higher frequency, with a total ESR of ∼96 Ω cm^2^. The ESR arises mainly from interfacial contacts, the polymer electrolyte matrix, active material and the electrode (graphite) sheet. Still, the values are comparable to those reported for many gel-based devices. The small semicircle indicates interfacial kinetics and arises from contributions from both bulk and interfacial charge transfer. The low-frequency vertical line represents the Warburg element due to charge accumulation at the electrode surface.^78^ This behaviour at a lower frequency (tail almost parallel to the vertical axis) indicates a predominantly capacitive response.

Fig. 9(d) shows the CV profile of the device over a potential window of up to 3 V at a scan rate of 100 mV s^−1^, measured at 40 °C. The plot shows that up to ∼2.25 V, the device exhibits good (box-like) CV behaviour, above which the CV begins to distort. The *C*_S_ at the inset is calculated from the CV curves across different potential windows. As the potential window (Δ*V*) increases, the area under the curve increases, leading to higher *C*_S_. A larger voltage window allows the ions to access more pores and penetrate deeper.

Fig. 9(e) shows the CV plots for the device over an operating potential window (*V*_op_) of 2 V at scan rates ranging from 10 to 100 mV s^−1^. At lower scan rates, the voltage changes slowly, allowing mobile ions sufficient time to diffuse into the larger and smaller pores of the AC. On the other hand, at high scan rates, the potential changes rapidly, so only the larger pores on the outer surface are accessible. Consequently, charge storage is limited at higher scan rates.^79^ The CV results are consistent, and the *C*_S_ monotonically decreases with increasing scan rate.

Fig. 9(f) shows the cycling stability of the device over 100 CV cycles at 2 V/100 mV s^−1^. The CV curves show no distortion, indicating excellent electrochemical stability and reversibility. Thus, the device can operate for an extended period without significant degradation.

**Fig. 10:**
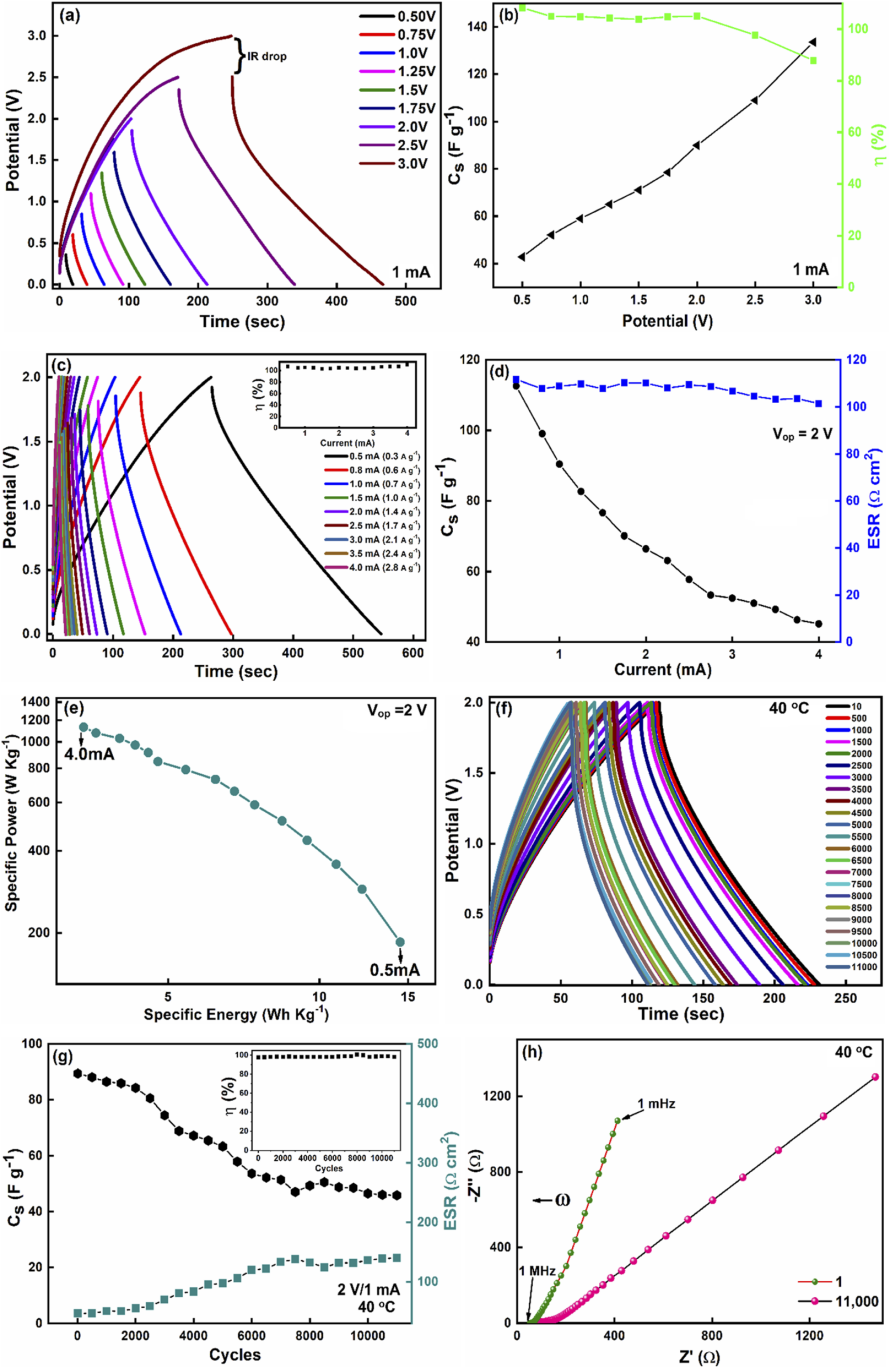
(a) GCD profile at different *V*_op_, (b) *C*_S_ and *η* variation over different *V*_op_, (c) GCD profile at different applied current, inset shows *η* with operating current, (d) *C*_S_ and ESR variation at different applied current, (e) Ragone plot (*E*_S_*versus P*_S_) at 2 V, (f) long cycling (11 000 GCD cycles) of device at 2 V/1 mA, (g) *C*_S_ and ESR variation over 11 000 GCD cycles, inset shows *η* variation with cycles, (h) Nyquist plot for SSC before and after GCD long cycles.

The galvanostatic charge–discharge (GCD) profile (at 1 mA) of the device at different cutoff voltages (Fig. 10(a)) is evaluated to determine the optimal operating potential window for suitable device performance. The GCD plots show that the charging–discharging behaviour is nearly triangular up to 2 V, resembling predominant EDLC behaviour, and beyond 2 V, a slight deviation from triangular shape. Apparently, the GCD profile indicates that 2 V is the optimal *V*_op_ for the best device performance.

Fig. 10(b) shows the variation of *C*_S_ and *η* with the operating potential (*V*_op_). As the *V*_op_ limit increases, the *C*_S_ increases monotonically as more ions are pulled towards the porous electrodes and more pores are utilized, resulting in higher energy storage and higher *C*_S_. For *V*_op_ ≤ 2 V, *η* slightly exceeds to 100%. This may be attributed to the surface complexity of AC electrodes. The AC material may trap some mobile ions during initial cycles and release them during subsequent discharge cycles. This delayed redistribution of ions results in additional discharge time.^80^ Beyond 2 V, *η* decreases due to increased ESR.

Fig. 10(c) shows the GCD profile at different currents ranging from 0.5 mA (0.3 A g^−1^) to 4.0 mA (2.8 A g^−1^) at a fixed *V*_op_ of 2 V, with efficiency close to 100%. As the current increases, ions are forced to move faster, resulting in interfacial and local polarization, and a high IR drop. Fig. 10(d) shows the variation of *C*_S_ and ESR with the discharge current. *C*_S_ decreases as the discharge current increases, while the ESR remains nearly 100 Ω cm^2^ throughout. At 2 V/1 mA (0.7 A g^−1^), the device delivers a *C*_S_ ∼90 F g^−1^.

Fig. 10(e) shows the Ragone plot for the device at 2 V. The device delivers a high *P*_S_ ∼1133 W kg^−1^ at 4.0 mA and a high *E*_S_ ∼15 Wh kg^−1^ at 0.5 mA. In bare interface conditions, supercapacitor formation is evident, with performance parameters close to some of the previously reported gel-based supercapacitors.^81–83^

Fig. 10(f) shows the long-cycle profile of the SSC at 2 V/1 mA. The device shows stable cycling up to 11 000 GCD cycles. Fig. 10(g) shows the variation in *C*_S_ and ESR over 11 000 GCD cycles, with *η* shown in the inset. The device shows ∼55% *C*_S_ retention after 11 000 cycles, with *η* >98% throughout cycling. Further, ESR varies from ∼48 Ω cm^2^ for the 1st cycle to ∼139 Ω cm^2^ for the 11 000th cycle. Fig. 10(h) shows the Nyquist plot of the SSC before and after long cycling, confirming stable performance.

Thus, bare SSCs demonstrated stable performance, exhibiting primarily EDLC-type behaviour for *V*_op_ ≤ 2 V. The electrode–electrolyte (solid–solid) interface was further tailored in order to enhance SSC performance.

#### 3.6.2 Interface-engineered SSCs

**Fig. 11:**
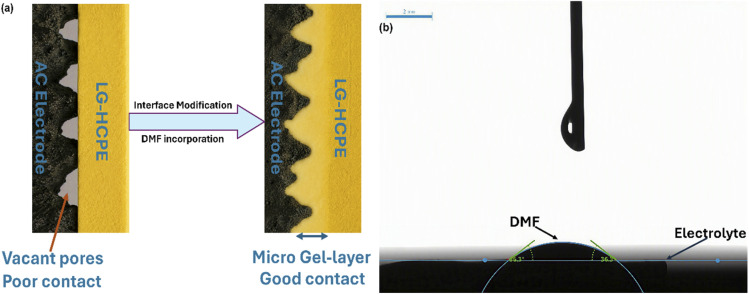
(a) Schematic representation of interface modification by the addition of DMF. A gel-layer connecting the electrode and electrolyte, (b) contact angle between DMF and composite polymer electrolyte.

Fig. 11(a) illustrates the electrode–electrolyte interface before and after adding DMF to the membrane surface. Voids at the interface due to poor contact leave pores in the AC electrodes unfilled, potentially reducing SSC performance. To address this, a small amount of DMF is applied to the surface of the solid polymer electrolyte to couple the interface. This results in a localized micro-gel layer forming at the interface, filling the empty voids in the surface of AC electrodes. Such an addition ensures better contact between the electrode and the electrolyte, as also reported by our group recently for NZSP-polymer composite-based SSCs.^84^

The contact angle measurement for DMF on the garnet-polymer membrane is shown in Fig. 11(b). The smaller the contact angle, the more uniformly the solvent spreads on the surface. The contact angle upon the addition of DMF is 36.3°, indicating that DMF spreads rapidly across the electrolyte surface. The identical angles on both sides can be attributed to the smooth electrolyte surface and uniform solvent spreading, which help create smooth contact between the electrode and the electrolyte, thereby increasing the effective utilization of the electrode surface area, reducing interfacial resistance, and improving ion transport.

**Fig. 12:**
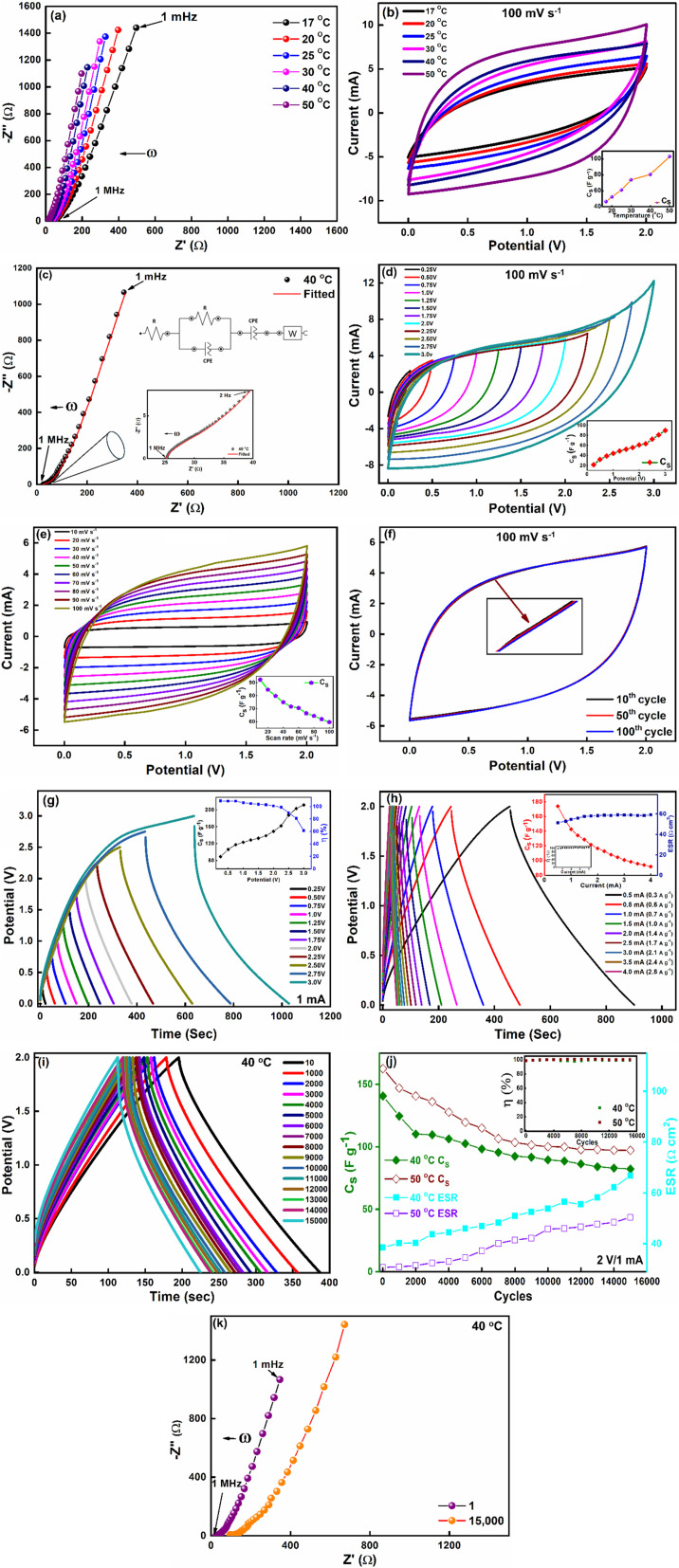
(a) EIS as a function of temperature, (b) CV profile as a function of temperature, inset shows the *C*_S_ (from CV) as a function of temperature, (c) fitted Nyquist plot of DMF incorporated SSC, inset shows the equivalent circuit and extended scale of high-frequency region, (d) CV plots at different *V*_op_, inset shows *C*_S_ (from CV) variation with potential, (e) CV at different scan rates, inset shows *C*_S_ (from CV) variation with scan rate, (f) CV 100 cycles at 2 V/100 mV s^−1^, inset shows zoom view of cycles, (g) GCD profile with different *V*_op_, inset shows *C*_S_ and *η* variation with potential, (h) GCD profile with different currents, inset *C*_S_ and ESR variation with current along with *η*, (i) 15 000 GCD long cycling, (j) *C*_S_ and ESR at 40–50 °C, inset shows *η* over 15 000 cycles, (k) EIS before and after 15 000 GCD cycles.

Fig. 12(a) shows the Nyquist plot of the device (DMF) as a function of temperature. The addition of DMF softens the electrolyte surface, improving contact at the electrode–electrolyte interface and thereby reducing the overall ESR. As discussed, as the device operating temperature increases, the thermally activated behaviour of the electrolyte increases, thereby enhancing electrical transport in the garnet-polymer membrane. The addition of DMF couples the electrode and electrolyte, allowing mobile ions to effectively access the surface area of AC. Subsequently, the addition of DMF helps the device to store more charge. This is confirmed by the CV profile of the device, as shown in Fig. 12(b). Where the CV plot exhibits a more rectangular shape and an increased area under the curve as temperature increases. Along with a significant increase in the *C*_S_ after the addition of DMF (inset).

Fig. 12(c) shows the Nyquist plot of the DMF device, with the inset showing the fitted circuit and the extended high-frequency region. The total ESR has decreased from ∼96 Ω cm^2^ for the bare SSC to ∼42 Ω cm^2^ for the DMF-incorporated SSC.

Fig. 12(d) shows the CV profile of the device up to 3 V, and the inset shows the change in the corresponding *C*_S_*versus V*_op_. Evidently, the area under the curve and the current increased drastically. The CV also shows a reasonably rectangular shape up to ∼2.5 V, reflecting enhanced capacitive behaviour and no evidence of chemical reaction.

The CV plots (Fig. 12(e)) at 2 V with different scan rates (10–100 mV s^−1^) were also obtained, and the inset shows *C*_S_ variation with scan rate. At low scan rates, the device exhibits high capacitive behaviour, which persists even at higher scan rates, indicating that the pores of the carbon electrode are well utilized by the electrolyte ions.

Fig. 12(f) shows the electrochemical stability of the device over the first 100 CV cycles at 2 V/100 mV s^−1^. The cycles are truly reproducible with negligible degradation in device performance, indicating good stability.

The GCD profile of the device at different *V*_op_ at 1 mA is shown in Fig. 12(g), and the inset shows the variation in *C*_S_ and *η* with potential. With the incorporation of DMF, not only did the time for a single GCD cycle improve, but the device also easily reached up to 3 V. A smooth interface apparently enhances ion participation in the electric double-layer formation process. Moreover, the IR drop reduced substantially, even at higher *V*_op_. The *C*_S_ has spiked to ∼212 F g^−1^ at 3 V/1 mA, compared to ∼134 F g^−1^ at 3 V/1 mA for the bare SSC.

Fig. 12(h) shows the GCD plot at different applied currents (0.5 mA to 4.0 mA), where the *V*_op_ for the GCD cycle is capped at 2 V, and the inset shows the *C*_S_ and ESR variation along with *η* over applied current. The GCD cycle for every applied current has significantly improved. The device delivers a high *C*_S_ ∼143 F g^−1^ (±8 F g^−1^) at 2 V/1 mA and a low ESR of ∼55 Ω cm^2^ compared to the bare device, which delivers *C*_S_ ∼90 F g^−1^ at 2 V/1 mA and ESR of ∼113 Ω cm^2^ (from GCD). This reduction in ESR and increase in *C*_S_ demonstrate that incorporating DMF (micro layer) significantly improves device performance parameters.

Fig. 12(i) shows the device up to 15 000 GCD cycles at 2 V/1 mA. The shape is almost triangular and is retained during long cycling. The variation of *C*_S_ and ESR with cycle number is shown in Fig. 12(j). The device shows *C*_S_ retention of ∼58% at 40 °C and ∼60% at 50 °C after long GCD cycles. The inset of the figure suggests that *η* remains >99% throughout cycling, and ESR changes from ∼38 to 66 Ω cm^2^ at 40 °C and from ∼30 to 50 Ω cm^2^ at 50 °C from 1st to 15 000th cycle. Furthermore, Fig. 12(k) shows the Nyquist plots of the device before and after 15 000 GCD cycles. Even after 15 000 GCD cycles, the device exhibits good capacitive behaviour.

Further, to compare bare and DMF-based SSCs, several parameters were evaluated. One can readily conclude that DMF at the interface improves the overall performance of SSCs.

The Bode analysis of SSCs (with and without DMF) reveals that the charge storage in these devices is predominantly EDLC-type. The addition of DMF not only improves ion transport across the interface but also reduces the relaxation time for the effective redistribution of charges (SI Fig. S3).

**Fig. 13:**
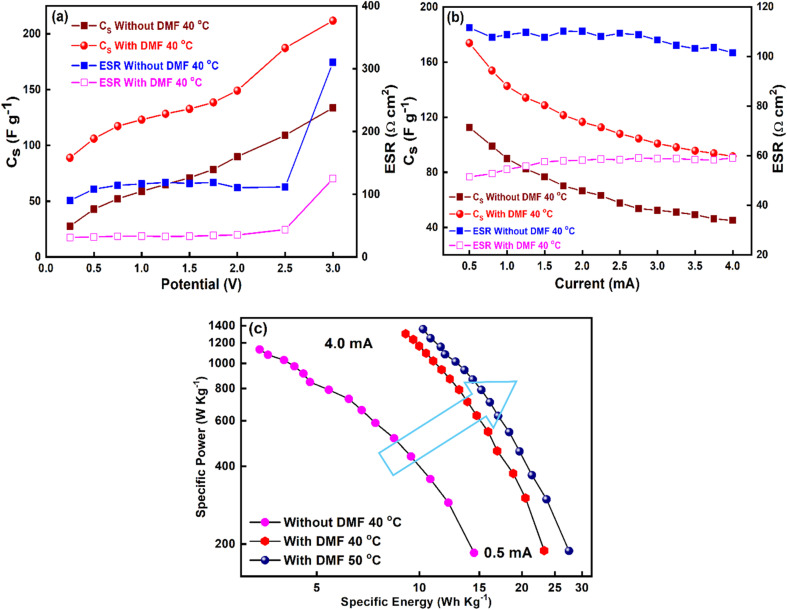
(a) Comparative plot of *C*_S_ and ESR with potential for the bare and the DMF device (at 1 mA), (b) comparative plot of *C*_S_ and ESR with current for the bare and the DMF device (at 2 V), (c) Ragone plot of bare device (40 °C) and DMF device (40 and 50 °C), arrow indicates improvement in performance, when DMF is added to the interface.

Fig. 13(a) shows the *C*_S_ and ESR variations with potential for both bare and DMF devices at 1 mA applied current. There is a notable increase in *C*_S_ and a significant decrease in ESR even at higher *V*_op_. Fig. 13(b) shows the variation of *C*_S_ and ESR over a 2 V potential window at different applied currents. Even at higher current, the DMF device shows excellent properties compared to the bare device.

Fig. 13(c) shows the Ragone plot for the bare device at 40 °C and the DMF device at both 40 °C and 50 °C. The incorporation of DMF also increased the specific energy and specific power of the device. Initially, the bare device delivers *E*_S_ ∼15 Wh kg^−1^ (at 0.5 mA), *P*_S_ ∼1133 W kg^−1^ (at 4.0 mA) at 40 °C, which is improved to *E*_S_ ∼24 Wh kg^−1^ (at 0.5 mA), *P*_S_ ∼1303 W kg^−1^ (at 4.0 mA) at 40 °C and *E*_S_ ∼28 Wh kg^−1^ (at 0.5 mA), *P*_S_ ∼1356 W kg^−1^ (at 4.0 mA) at 50 °C.

Fabricating an energy storage device with both high *E*_S_ and *P*_S_ simultaneously is the ultimate goal in modern energy storage systems, and utilizing the potential of Li^+^-garnet with interface engineering can help achieve this goal.

It may be summarized here that the addition of DMF forms a micro-gel layer (as in Fig. 5(d)) at the electrode–electrolyte interface, reducing interfacial resistance. The DMF-incorporated device shows a significant increase in performance. This can be due to several reasons.

(i) The solvent layer helps in creating good contact between the electrode and the electrolyte, which helps in reducing ESR and enhances the utilization of the pores on the surface of the AC electrode. The DMF has a high dielectric constant, which can enhance ion dissociation as the interaction between cations and anions decreases, resulting in increased free charge carriers and improved Li^+^-ion mobility.

(ii) DMF has low viscosity, and ions can rearrange quickly, which helps in forming the electric double layer more effectively.

(iii) DMF spreads rapidly and evenly across the electrolyte surface as suggested by contact angle measurements.

#### 3.6.3 Temperature tolerance

**Fig. 14:**
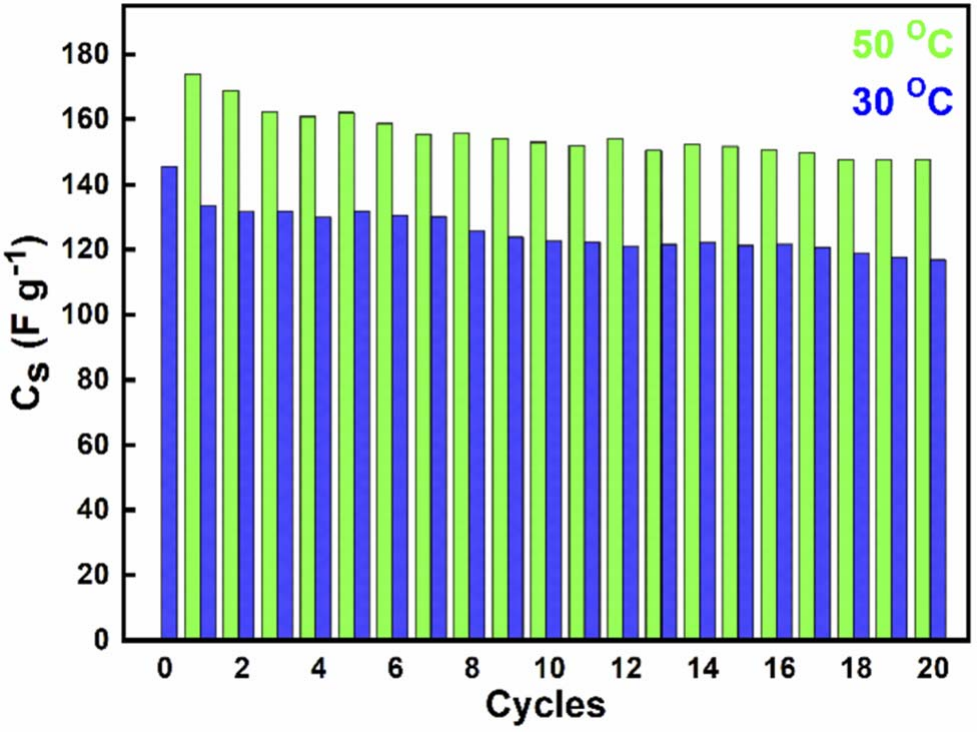
Change in *C*_S_ with variable operating temperature during 20 iterations (2 V/1 mA) between 30 and 50 °C.

The thermal stability of the cell under varying working temperatures was tested by performing 40 GCD cycles at 2 V/1 mA between 30 °C and 50 °C (20 consecutive measurements at each temperature after cycling), during which a change in *C*_S_ was monitored (Fig. 14). Even after 20 iterations, the SSC performs quite well and shows no sign of any major degradation, indicative of stable performance.

Furthermore, to assess the wide operating temperature range of the SSCs, the device performance was also tested below ambient temperature. The low-temperature studies of the SSCs not only demonstrate their potential to operate under extreme conditions but also indicate stable performance even after operating below 0 °C, as expected from a reliable energy storage device.

**Fig. 15:**
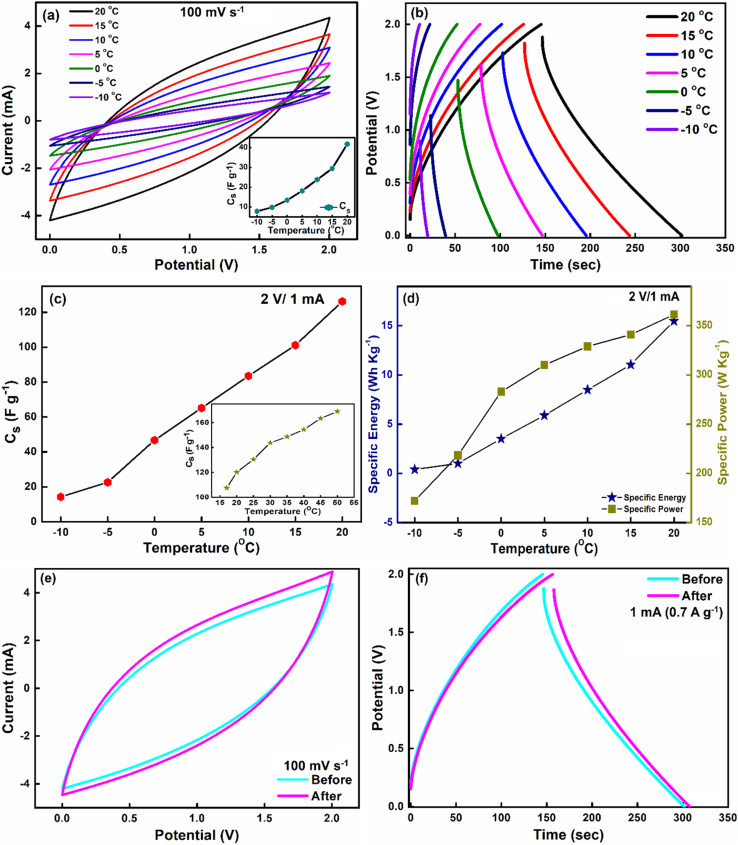
(a) CV profile as a function of temperature, inset shows *C*_S_ (from CV) variation with temperature, (b) temperature dependent GCD plots, (c) *C*_S_ variation over ambient operating temperature, inset shows *C*_S_ (from GCD) variation above room temperature, (d) *E*_S_ and *P*_S_ correlation with operating temperature, (e) and (f) CV and GCD plots at 20 °C before and after cycling up to −10 °C.

Fig. 15 illustrates the low-temperature device performance. In Fig. 15(a), the CV profile is shown as a function of temperature, with the inset showing the *C*_S_ variation with temperature. As expected, when the temperature is lowered, the area under the CV curves decreases, indicating reduced charge storage due to decreased ion mobility.

The GCD plots were recorded at different operating temperatures (Fig. 15(b)). As temperature decreases, relatively fewer ions participate in charge storage due to reduced thermal activation, and the electrolyte becomes stiffer, leading to poor interfacial contact. The time per GCD cycle also decreases, and ESR increases.

Fig. 15(c) shows *C*_S_ variation as the temperature varies from −10 °C to 20 °C, while the inset shows *C*_S_ variation between 17 °C and 50 °C. Even below 0 °C, the SSC shows adequate performance, making it useful for low-power applications. This highlights the excellent compatibility of Li^+^-garnet polymer composite for electrolytic usage in SSCs. As the temperature decreases, the concentration of mobile ions decreases, resulting in lower ionic conductivity. The contact resistance between the electrode and the electrolyte increases, resulting in an increased IR drop and reducing the discharge time. The devices are well capable of operating effectively down to −10 °C, below which ion transport is insufficient to facilitate energy storage, as discussed in Section 3.5. The inset shows that *C*_S_ increases gradually from ∼107 F g^−1^ at 17 °C to ∼169 F g^−1^ at 50 °C. As temperatures rise, thermally activated behaviour revokes, and gradually more Li^+^-ions become mobile to participate in charge storage.

Fig. 15(d) shows the variation of *E*_S_ and *P*_S_ with temperature. At lower temperatures, interfacial contact is poor, and ion dynamics and diffusion are slow, so fewer ions participate in charge storage. Resulting in lower values of *E*_S_ and *P*_S_.

Fig. 15(e) and (f) shows the CV and GCD plots during cycling between 20 °C and −10 °C. The plots again indicate that even at low temperatures, the device still operates normally, demonstrating its stability and reproducibility.

#### 3.6.4 SSC under prolonged GCD cycling

**Fig. 16:**
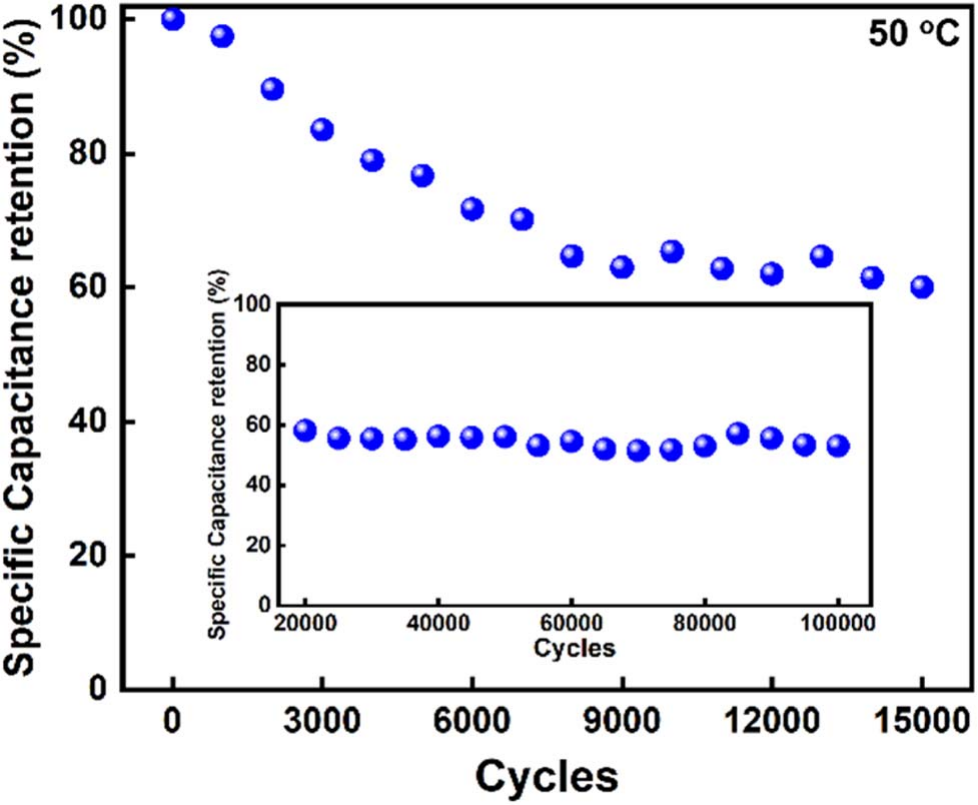
100 000 GCD cycles of the SSC at 2 V/1 mA at 50 °C, the inset shows stable behaviour between 20 000 and 100 000 GCD cycles.

To predict the long-term electrochemical stability and operational lifespan, prolonged GCD cycling was performed for these garnet-polymer composite-based SSCs. The SSC was tested for 100 000 GCD cycles (50 °C) at 2 V/1 mA and demonstrated remarkably stable performance even after 100 000 cycles (Fig. 16). The *C*_S_ value fell initially and stabilized subsequently after ∼20 000 cycles to the same value for the rest of 80 000 cycles (inset). This suggests the formation of stable ion conduction pathways in the garnet-polymer matrix and stabilization of the electrode–electrolyte interface. Interestingly, the SSC exhibits a *C*_S_ retention of ∼53% after 100 000 cycles, along with *η* ∼100%. The potential of the Li^+^-garnet polymer-based device for long-term power delivery is thus witnessed (Table 1).

**Table 1:** A comparison of the present study with previous investigations on gel polymer electrolyte (GPE) and solid polymer electrolyte (SPE)-based supercapacitors

S. No	Electrolyte	Electrode	*σ*_DC_ (Ω^−1^ cm^−1^)	*C*_S_ (F g^−1^)	*E*_S_ (Wh kg^−1^)	*P*_S_ (W kg^−1^)	Device performance	Ref.
1	LATP composite with LiClO_4_ and PEO	AC	1.6 × 10^−4^ (40 °C)	100 (2 V/1 mA)	14 (0.4 mA)	682 (4.0 mA)	∼25% retention after 2500 cycles	72
2	NZSP composite with NaCF_3_SO_3_ and PEO	AC	2 × 10^−4^ (40 °C)	150 (1 V/2 A g^−1^)	3.55 (1 mA)	607 (1 mA)	∼93% retention after 2500 cycles	74
3	NZSP composite polymer electrolyte	AC	2.7 × 10^−4^ (40 °C)	260 (2 V/1 mA)	32 (1 mA)	787 (1 mA)	∼60% retention after 10 000 cycles	57
4	PCL : PVDF-HFP activated with 1 M NaClO_4_ in EC : PC	ZnAC	1.5 × 10^−3^ (room temperature)	247.82 (2 V/0.52A g^−1^)	22.58 (0.52 A g^−1^)	420 (0.52 A g^−1^)	∼97% retention after 10 000 cycles	85
5	SPE of PLI-PP with 70 wt% IL	AC	2.9 × 10^−3^ (25 °C)	158 (2.5 V/0.1 A g^−1^)	13.8 (1 A g^−1^)	1060 (1 A g^−1^)	90% retention after 1000 cycles	86
6	Epoxy-based SPE with IL (50) and Al_2_O_3_	AC	2.9 × 10^−4^ (25 °C)	90 (2.5 V/0.13 A g^−1^)	75 (1 A g^−1^)	382 (1 A g^−1^)	61% retention after 1000 cycles	87
7	Na-GPE with IL in 0.5 M NaBF_4_	AC	8.7 × 10^−3^ (30 °C)	257 (1 V/1 mA cm^−2^)	9 (1 mA cm^−2^)	100–900	79% retention after 8000 cycles	88
8	PVdF-HFP/ADN/LiTFSIGPE	AC	1.97 × 10^−3^ (25 °C)	225–241 (2 V/1 mA cm^−2^)	31.2–34.0 (1 mA cm^−2^)	15 400–18000 (1 mA cm^−2^)	∼80% retention after 20 000 cycles	89
9	SPE with 45LLZNO12LiClO_4_	AC	2 × 10^−4^ (30 °C)	143 (2 V/1 mA)	24 (0.5 mA)	1303 (4.0 mA)	∼60% retention after 15 000 cycles	Present work
							∼53% after 100 000 cycles	

## 4 Charge storage mechanism

The CV, GCD cycles, and EIS spectra suggest that the charge storage is essentially of an electric double-layer type, and that creating a gel layer at the interface does not alter the charge storage mechanism in these SSCs. The performance, though, improves after the gel layer incorporation.

Dunn's method is used to further assess the charge-storage mechanism (capacitive *versus* diffusive), and the analysis is provided in the supplementary. Quantitatively, the charge-storage mechanism remains surface-controlled, and no noticeable reaction is witnessed at the interface.

**Fig. 17:**
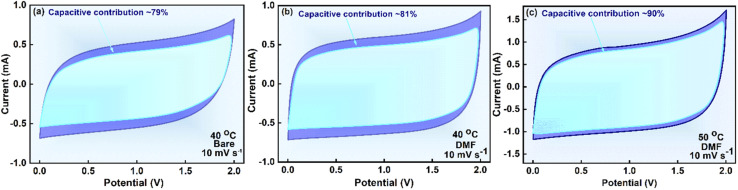
Dunn's plot of (a) Bare device at 40 °C, (b) DMF device at 40 °C, (c) DMF device at 50 °C.

It is found that the charge storage mechanism in the fabricated SSCs is capacitive-dominated. This confirms the predominant EDLC behaviour of the SSC. Even the bare SSC shows ∼79% capacitive charge storage at 40 °C, indicating faster ion kinetics near the surface of the electrode and the electrolyte, as shown in Fig. 17(a). The improved interfacial contact after the addition of DMF enhances EDLC behaviour by increasing the capacitive contribution to ∼81% at 40 °C, as shown in Fig. 17(b). As device operating temperature increases, internal resistance decreases, and ion mobility/transport increases, which further enhances the capacitive behaviour to ∼90% (Fig. 17(c)).

## 5 Practical uses of LG-HCPE SSC cells

**Fig. 18:**
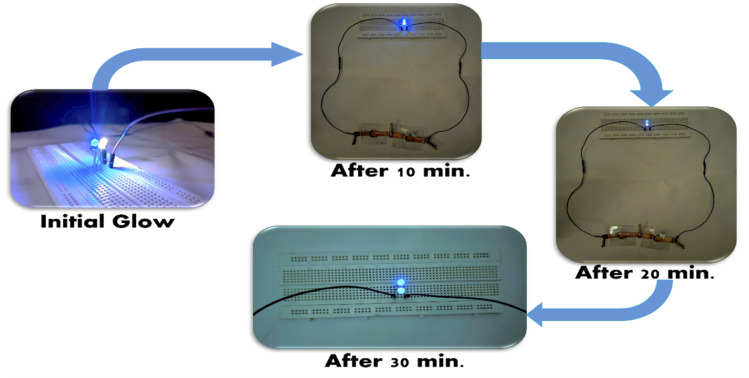
Demonstration of real-life application of the device by glowing two LEDs (3 V each) connected in parallel. Only two SSCs (2 V each) are sufficient for such usage.

Finally, to demonstrate the real-life application of SSC, a blue (3.2 V) and a white (3.2 V) colour LEDs were connected in parallel, and two SSCs were connected in series. The combination glows the LEDs for more than 30 minutes (Fig. 18). This further highlights their potential in energy storage devices.

Garnet-polymer composites-based SSCs thus exhibit a wider temperature tolerance, long cycling, and better performance than previously investigated NASICON-polymer,^74^ LATP-polymer,^72^ and LTP-polymer^69^ based SSCs.

## 6 Conclusions

In the present work, to the best of our knowledge, a Li^+^-garnet hybrid composite polymer has been used for the first time as an electrolyte in solid-state supercapacitors.

For this, Cubic LLZNO was synthesized *via* the solid-state method, where the Cubic phase formation is verified from XRD and Rietveld analysis. The addition of salt (LiClO_4_) and LLZNO reduces the crystallinity of PEO, thereby improving ion transport, as confirmed by FTIR, Raman, and DSC analysis. The optimized LG-HCPE (45LLZNO_12S) solid polymer electrolyte exhibits excellent properties, with a high ionic conductivity of ∼2 × 10^−4^ Ω^−1^ cm^−1^ at 30 °C. The bare SSC device (without interface modifications) exhibits good and stable performance. After interface modification, the device delivers *C*_S_ ∼143 F g^−1^ at 2 V/1 mA, with cycling stability up to 15 000 GCD cycles while retaining ∼60% *C*_S_ and can be operated for up to 100 000 GCD cycles. The SSC can work effectively below 0 °C with excellent reversibility. Further device stability is assessed by performing 20 GCD iterations at 30 and 50 °C. Results suggest that charge storage is predominantly capacitive, which is a key characteristic of EDLCs, and is further improved by the addition of DMF. In fact, polymer-garnet-based devices may be a better choice for EDLC fabrication than the other ceramic-in-polymer-based SSCs.

Finally, the garnet-based hybrid electrolyte holds significant promise for future sustainable energy storage solutions. While Li^+^-ion garnet offers extensive electrochemical stability, its application in EDLCs is promising, but its potential can be further utilized. These materials should be explored for the development of high-performance pseudo-capacitors and hybrid devices, *a* direction we are actively pursuing.

## Author contributions

Anshuman Dalvi: conceptualization, validation, writing – review and editing, funding acquisition, Kamaldeep Bisht: conceptualization, methodology, validation, formal analysis, investigation, writing – original draft, Bhargab Sharma: methodology, Hardeep: formal analysis, Arpita Mishra: formal analysis.

## Conflicts of interest

The authors declare no conflict of interest.

## Supplementary Material

RA-OLF-D6RA05078F-s001

## Data Availability

All relevant data are available from the corresponding authors upon reasonable request. Supplementary information (SI) is available. See DOI: https://doi.org/10.1039/d6ra05078f.
