# Supplementary material for: Enhancing the performance of solid-state supercapacitors using Li+-garnet polymer hybrids as electrolyte
Source: RSC Adv. 2026 Aug 10;16(42):50081–105. doi: 10.1039/d6ra05078f (PMC13456103; doi:10.1039/d6ra05078f)
Supplement: Supplementary file 1 [file RA-016-D6RA05078F-s001.pdf]

Supplementary Information (SI) for RSC Advances.
This journal is © The Royal Society of Chemistry 2026

Enhancing performance of solid-state supercapacitors using Li^+-garnet polymer hybrids as electrolyte

Kamaldeep Bisht, Bhargab Sharma, Hardeep, Arpita Mishra and Anshuman Dalvi*

Department of Physics, Birla Institute of Technology and Science, Pilani, Pilani Campus, Vidya Vihar, Pilani, Rajasthan 333031, India

*Corresponding Author E-mail: adalvi@pilani.bits-pilani.ac.in

1. Thermogravimetric analysis of the electrolyte membrane (TGA)

The thermogravimetric analysis (Fig. S1) of the composite electrolytes was performed to assess the composite polymer electrolyte's resistance to decomposition. The pristine PEO and the corresponding composite polymer electrolyte remain nearly stable until ~300 °C and exhibit major decomposition between 300 °C and 400 °C.[1,2] An initial weight loss of ~4% was observed up to ~100 °C for all the compositions except PEO, which may be attributed to the loss of moisture that arises due to the hygroscopic nature of the salt $LiClO_4$ and Li^+-garnet (LLZNO). As LLZNO replaces PEO content in the electrolyte, the gradually higher residual weight is mainly attributed to the presence of LLZNO in increasing amounts in the matrix. As is evident (inset), the thermal stability limit of the electrolyte is further extended by the addition of LLZNO garnet. This may again be attributed to the relatively low presence of PEO in the matrix.

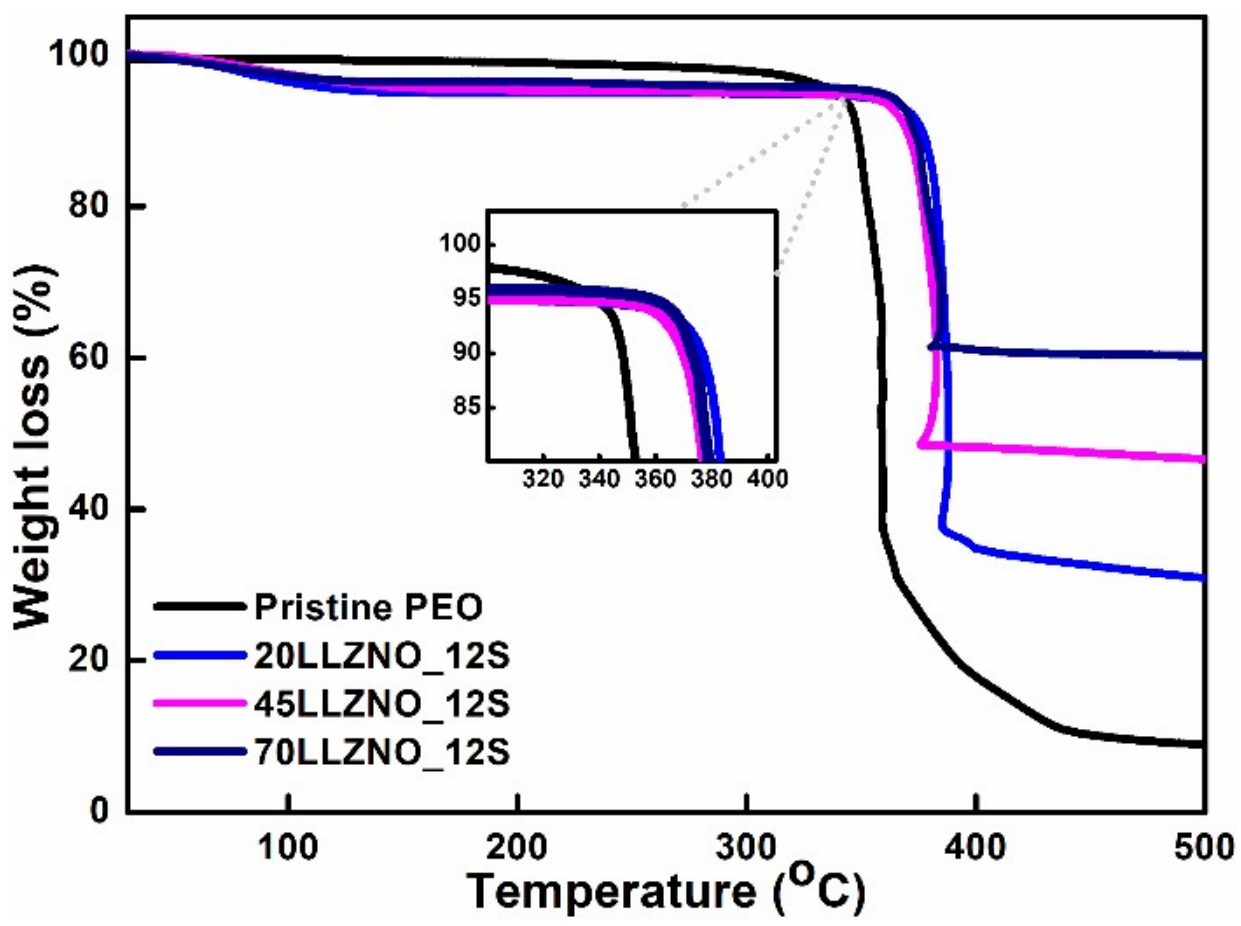

Fig. S1 TGA analysis of garnet-polymer composite with xLLZNO content

2. Measurement of Ionic transference number

The predominant ionic transport in these composites is supported by measurements of the ionic transference number using the symmetric Hebb-Wagner DC polarization method.[3] For this, the synthesized LG-HCPE is placed between blocking stainless steel (SS) electrodes, a DC potential of 0.5 V is applied, and the transient DC current is monitored as a function of time. The ionic transference number is calculated using the following relation

$$t_i = 1 - \left(\frac{I_s}{I_o}\right)$$

where I_S and I_o represent the saturation current and the peak currents, respectively. Notably, t_i is approximately 0.998, which indicates that electronic conductivity is negligible and mobile ions dominate the electrical transport.

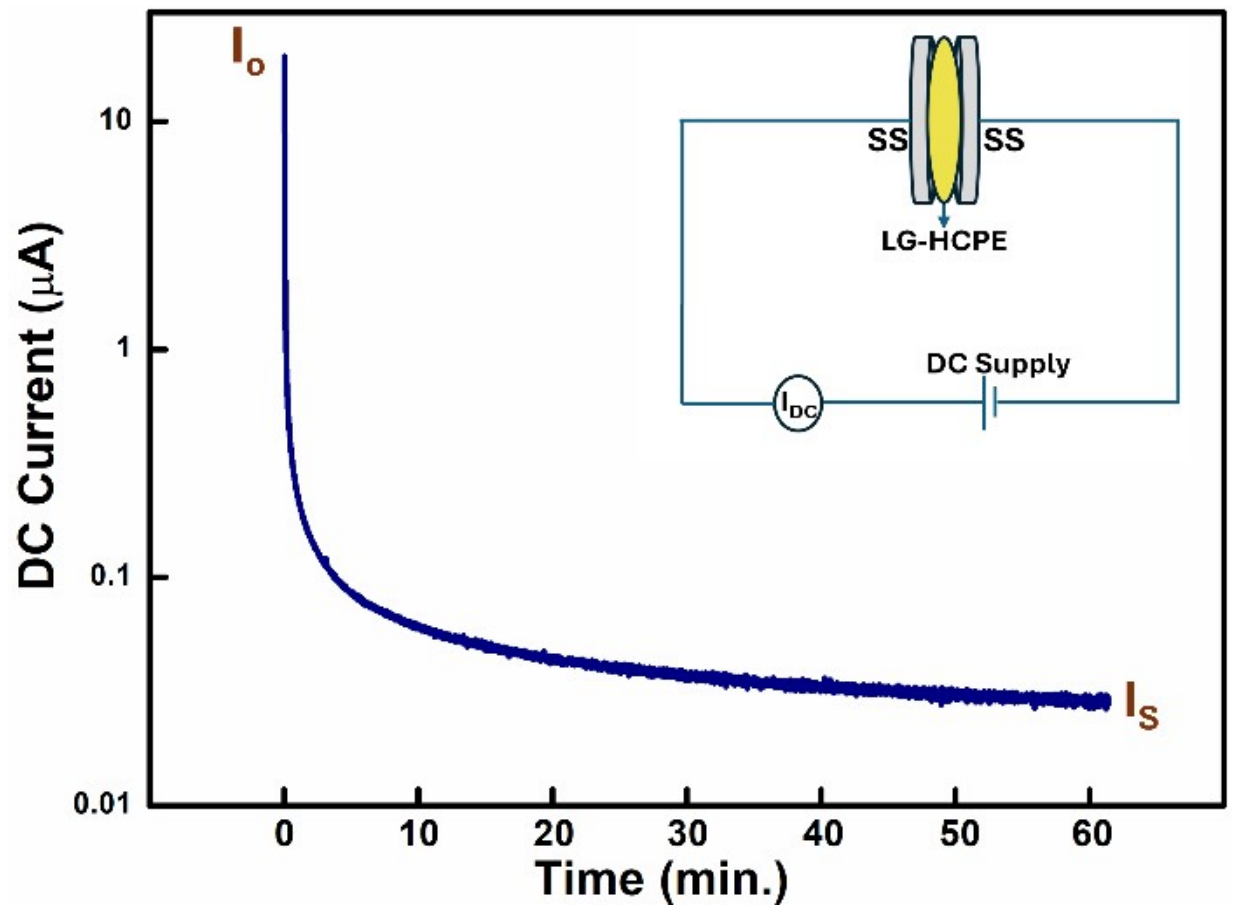

Fig. S2 DC current as a function of time from symmetric Hebb-Wagner DC polarization for ion transport measurement of LG-HCPE, the inset shows the circuit configuration.

3. Bode analysis

The Bode analysis was conducted to examine how the interfacial charge kinetics in the SSCs respond to time-varying external electric fields.

The phase angle-frequency plot (Fig. S3 (a)) describes the charge storage mechanism in a supercapacitor. For an ideal EDLC, where a purely electrostatic mechanism of charge storage predominates, the phase angle approaches -90 ° at low frequencies.[4] It is evident that, even before interface modification, the device exhibits a reasonable EDLC behaviour with a low frequency phase angle of ~ -75 °, which is further enhanced upon the addition of DMF at the interface, approaching ~ -80 °.

The characteristic frequency (f_o) at -45 ° (shown by a dotted line) infers the transition between the resistive and capacitive behaviour of the SSC.[5] For bare device f_o ~29 mHz and after the

incorporation of DMF f_o shifts to a higher frequency at ~78 mHz, which indicates the presence of DMF leads to substantial improvement in ion transport across the interface.

As shown in Fig. S3 (b), at higher frequencies, the device impedance decreases significantly upon the addition of DMF, thereby facilitating faster ion transport. This suggests that ions quickly access the electrode pores, resulting in high performance parameters.[6]

The imaginary capacitance $C'' = \frac{Z'}{\omega|Z|^2}$ also provides important insight into charge storage at the interface, as shown in Fig. S3 (c). The plot of C'' versus frequency shows the relaxation frequency (shown by a dotted line) at which C'' is maximum.[7,8] Evidently, even before the DMF layer, the relaxation frequency (f_c) is adequate for faster kinetics. After DMF incorporation, the peak slightly shifts toward a higher frequency, indicating faster ion mobility and improved rate capability. The relaxation peak in the bare system arises at ~8 mHz, with a relaxation time constant (τ_o) ~125 sec. In comparison, after the addition of DMF, f_c was observed at ~27 mHz with τ_o ~37 sec, indicating a faster process for the effective redistribution of charges. This also suggests improvement in the power capability of the supercapacitor.

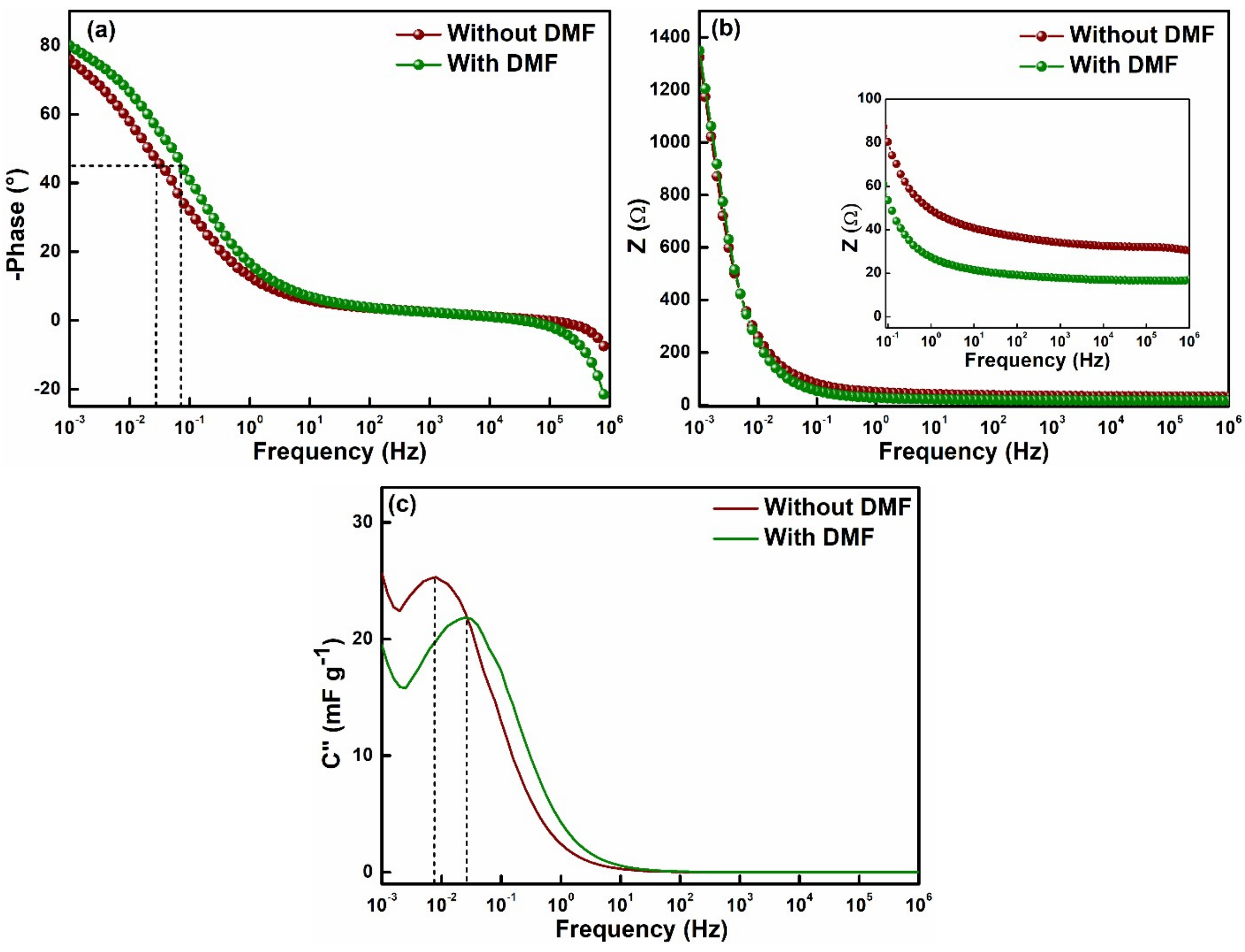

Fig. S3 Bode analysis of SSCs before and after the addition of DMF. (a) Phase variation as a function of frequency, (b) Impedance variation with frequency, inset shows the zoomed view of the high frequency region, (c) Variation of imaginary capacitance (C'') versus frequency.

4. Dunn's method

The quantitative analysis of the charge storage mechanism in the fabricated SSCs is done using Dunn's method. For this CV, plots were obtained at different scan rates (1–10 mV s^{-1}).[9,10]

The direct relation between the peak current and scan rate is

$$i_p = av^b$$

Where i_p is the peak current, v is the scan rate, a (y-intercept) & b (slope) are the constants describing the nature of the charge storage mechanism. For the capacitive process, b value approaches unity, for the diffusive process, it remains near 0.5, and 0.5 < b < 1 for the mixed mechanism.

The peak current for a supercapacitor with a mixed charge storage mechanism (as observed in most of the cases) is given by:

$$i_p = k_1 v + k_2 v^{\frac{1}{2}}$$

Where i_p is the peak current, k_1 (slope) and k_2 (y intercept) are the constants from the $i(v)/v^{\frac{1}{2}}$ vs $v^{\frac{1}{2}}$ plot. The capacitive contribution is drawn from the first term $k_1 v$ while the second term $k_2 v^{\frac{1}{2}}$ gives the diffusive contribution to the total peak current. The values of the constants and the current contribution are calculated from the CV plots.

References

1. Li R, Guo S, Yu L, Wang L, Wu D, Li Y, Hu X, Morphosynthesis of 3D Macroporous Garnet Frameworks and Perfusion of Polymer-Stabilized Lithium Salts for Flexible Solid-State Hybrid Electrolytes, *Adv Mater Interfaces*, 2019, **6(10)**, doi:10.1002/admi.201900200.

2. Pandurangan I, Sathiasivan K, Aarimuthu G, Kannadasan M, Balakrishnan M, Lithium perborate-based composite polymer electrolytes for all-solid-state lithium-ion batteries: performance enhancement and stability, *Ionics (Kiel)*, 2024, **30(8)**, 4531-4540, doi:10.1007/s11581-024-05641-y.

3. Bhardwaj P, Neha, Dalvi A, Sarkar M, Role of Sulfonamide-Based Covalent Organic Frameworks (COFs) in Enhancing the Electrochemical Performance of PEO-Based Composite Polymer Electrolytes, *ACS Appl Energy Mater*, 2025, **8(13)**, 9230-9243, doi:10.1021/acsaem.5c00849.

4. Xu Y, Wang S, Peng H, Yang Z, Martin DJ, Bund A, Nanjundan AK, Yamauchi Y, Electrochemical Characteristics of Cobaltosic Oxide in Organic Electrolyte According to Bode Plots: Double-Layer Capacitance and Pseudocapacitance, *ChemElectroChem*, 2019, **6(9)**, 2456-2463, doi:10.1002/celc.201900289.

5. Daraghmeh A, Hussain S, Saadeddin I, Servera L, Xuriguera E, Cornet A, Cirera A, A Study of Carbon Nanofibers and Active Carbon as Symmetric Supercapacitor in Aqueous Electrolyte: A Comparative Study, *Nanoscale Res Lett*, 2017, **12(1)**, 639, doi:10.1186/s11671-017-2415-z.

6. Silva BPG, Tosco B, de Florio DZ, Stepanenko V, Würthner F, Rey JFQ, Brochsztain S, Efficient Electronic Coupling in Perylenediimide Multilayered Films on Indium Tin Oxide, *The Journal of Physical Chemistry C*, 2020, **124(10)**, 5541-5551, doi:10.1021/acs.jpcc.9b11373.

7. Deshmukh AD, Urade AR, Nanwani AP, Deshmukh KA, Peshwe DR, Sivaraman P, Dhoble SJ, Gupta BK, Two-Dimensional Double Hydroxide Nanoarchitecture with High Areal and Volumetric Capacitance, *ACS Omega*, 2018, **3(7)**, 7204-7213, doi:10.1021/acsomega.8b00596.

8. Bello A, Manyala N, Barzegar F, Khaleed AA, Momodu DY, Dangbegnon JK, Renewable pine cone biomass derived carbon materials for supercapacitor application, *RSC Adv*, 2016,**6(3)**, 1800-1809, doi:10.1039/C5RA21708C.

9. Rafique A, Naeem U, Marques A, Ferreira I, Rizwan S, Baptista AC, Understanding the Capacitive and Diffusion-Controlled Behavior of Electrophoretically Deposited V_2CT_x on Carbon Yarn as a Potential Anode for Asymmetric Devices, *ACS Omega*, 2025, **10(8)**, 7621-7634, doi:10.1021/acsomega.4c06035.

10. Harish S, Sathyakam PU, Dunn's Method for Distinguishing Charge Storage Mechanisms in Supercapacitors: A Status Quo Review, *J Electron Mater*, 2025, **54(12)**, 10858-10872, doi:10.1007/s11664-025-12481-7.
